# Oxygen Sensing, Hypoxia Tracing and *in Vivo* Imaging with Functional Metalloprobes for the Early Detection of Non-communicable Diseases

**DOI:** 10.3389/fchem.2018.00027

**Published:** 2018-02-23

**Authors:** Vincenzo Mirabello, Fernando Cortezon-Tamarit, Sofia I. Pascu

**Affiliations:** Department of Chemistry, University of Bath, Bath, United Kingdom

**Keywords:** oxygen sensing, molecular imaging, hypoxia, metals in medicine, FRET

## Abstract

Hypoxia has been identified as one of the hallmarks of tumor environments and a prognosis factor in many cancers. The development of ideal chemical probes for imaging and sensing of hypoxia remains elusive. Crucial characteristics would include a measurable response to subtle variations of pO_2_ in living systems and an ability to accumulate only in the areas of interest (e.g., targeting hypoxia tissues) whilst exhibiting kinetic stabilities *in vitro* and *in vivo*. A sensitive probe would comprise platforms for applications in imaging and therapy for non-communicable diseases (NCDs) relying on sensitive detection of pO_2_. Just a handful of probes for the *in vivo* imaging of hypoxia [mainly using positron emission tomography (PET)] have reached the clinical research stage. Many chemical compounds, whilst presenting promising *in vitro* results as oxygen-sensing probes, are facing considerable disadvantages regarding their general application *in vivo*. The mechanisms of action of many hypoxia tracers have not been entirely rationalized, especially in the case of metallo-probes. An insight into the hypoxia selectivity mechanisms can allow an optimization of current imaging probes candidates and this will be explored hereby. The mechanistic understanding of the modes of action of coordination compounds under oxygen concentration gradients in living cells allows an expansion of the scope of compounds toward *in vivo* applications which, in turn, would help translate these into clinical applications. We summarize hereby some of the recent research efforts made toward the discovery of new oxygen sensing molecules having a metal-ligand core. We discuss their applications *in vitro* and/or *in vivo*, with an appreciation of a plethora of molecular imaging techniques (mainly reliant on nuclear medicine techniques) currently applied in the detection and tracing of hypoxia in the preclinical and clinical setups. The design of imaging/sensing probe for early-stage diagnosis would longer term avoid invasive procedures providing platforms for therapy monitoring in a variety of NCDs and, particularly, in cancers.

## Introduction

Non-communicable diseases (NCDs) account for over two thirds of annual deaths worldwide, reaching epidemic proportions and imposing a significant burden to all public health systems and economies. NCDs have increased substantially in the past decade with respect to communicable diseases, being the majority of premature deaths related to NCDs particularly occurring in low and middle income countries (Lozano et al., 2012).

International efforts and collaboration between countries and organizations were deemed necessary in the development of efficient detection, screening and treatment strategies to address NCDs according to the World Health Organization (WHO)^1^ (Daar et al., 2007). In the mid-term global action plan to reduce the occurrence of these diseases, the WHO identified several action points which include engaging with leaders, strengthening health systems, modifying unhealthy behaviors, encouraging research and monitoring progress to prevent and control the proliferation of NCDs (Daar et al., 2007)^1^.

The main contributors to NCDs burden in terms of mortality numbers are ischemic heart disease and cancers, which contribute to more than two thirds of the deaths which occur worldwide (Lozano et al., 2012; Bray et al., 2013). The detection of reduced oxygen levels in tissues helps when combating both NCDs by providing an early stage detection, a monitored progression through oxygen partial pressure (pO_2_, expressed in mmHg) sensing followed by an appropriate tailor-made treatment for the disease as hypoxia is one of their defining parameters. Hypoxia targeting is crucial in most solid tumors where there is an imbalance between the levels of O_2_ supply and consumption. This imbalance is a result of the uncontrolled cell proliferation and disorganized vasculature structure that is linked to poor blood supply causing areas with reduced pO_2_. Similarly, reduced oxygen levels develop in cardiac and brain ischemia, or after a stroke, due to the interruption of the blood supply (Giordano, 2005).

In particular, hypoxia has been identified as a hallmark of tumor environment and a fundamental prognostic factor in many cancers (Challapalli et al., 2017) therefore the development of a probe with increased response to subtle variations of pO_2_, increased kinetic stability which is able to accumulate only in the areas of interest, avoiding the necrotic or normoxic tissues, as well as the ability to provide structural and functional information of cells would be highly desirable. Such sensitive probes would contribute toward the design and delivery of a new platform for applications in imaging and therapy relying on sensitive detection of pO_2_ in cancers or other diseases. This would allow for the stratification of patients according to the severity of hypoxia levels and the development of personalized medicine strategies which can tailor radio- and chemotherapy treatments. The design of such an imaging/sensing probe would avoid invasive procedures and provide easy follow up of the disease during and after treatment.

The probe in question, unfortunately, to date remains to be discovered and validated, and thus far, only a handful of probes for the imaging of hypoxia [mainly using positron emission tomography (PET)] have reached clinical validation stages. They all still present considerable disadvantages for their general application, e.g., non-optimal pO_2_ response, lack of effectiveness for all tumors or high non-specific uptake. Furthermore, their mechanism of action has not been entirely rationalized hence the addition of multimodal properties including fluorescent groups to follow the probes *in vitro* prior to application *in vivo*/clinical would assist in elucidating the uptake mechanisms and mechanisms of action under different redox and pH environments. New insights into the elucidation of hypoxia selectivity mechanisms would allow for the optimization probe candidates, expanding the scope of compounds tested and helping to translate and adapt to *in vivo*, preclinical and clinical applications and the most up-to-date aspects will be highlighted hereby.

We summarize herein the current research efforts available in the public domain toward designing and testing oxygen responsive molecules and the application of fluorescence detection for characterization *in vitro* or *in vivo* thanks to near-infrared (NIR) emission. This is followed by a summary of the molecular imaging techniques applied in the detection and measurement of hypoxia in the preclinical and clinical setup and which rely frequently on nuclear medicine techniques such as positron emission tomography (PET) and single-photon emission computed tomography (SPECT).

### Sensing and imaging under O_2_ concentration gradients

Molecular oxygen, O_2_, is essential (Semenza, 2012) for the metabolism of aerobic organisms (Yoshihara et al., 2017) and plays a crucial role in the transformation of the energy stored in organic molecules in the high-energy phosphate bonds of adenosine triphosphate (ATP). Aerobic organisms consume fatty acid, amino acids and saccharides as metabolic “fuel” thus producing nicotinamide adenine dinucleotide (NADH) as well as flavin adenine dinucleotide (FADH_2_) (Giordano, 2005). Such high-energetic enzymatic cofactors are responsible for the generation of ATP molecules from ADP and enter the electron transfer cycles during oxidative phosphorylation processes. O_2_ is the terminal electron acceptor in the cellular mitochondrial respiratory chain (Semenza, 2007). The intracellular O_2_ concentration is regulated by a complex microcirculation system. Acute levels of increased or decreased cellular O_2_ concentrations (hyperoxia, and hypoxia respectively) generate an excess of reactive oxygen species (ROS). ROS may damage DNA activating protein p53 and Poly (ADP-ribosyl)ation (PARP), which, in turn, triggers the activation of protease (calpains), protein degradation, disruption of mitochondrial functions, thus lowering ATP levels and eventually leading to the cellular necrosis (Ame et al., 2004; Peixoto et al., 2017). In humans, it has been acknowledged that deregulated ROS can cause cellular dysfunction resulting in the development of common features that are typical of many NCDs including cancers, cardiovascular diseases, stroke and ultimately death (Semenza, 2012). Hypoxia is a characteristic feature of advanced solid tumors and, upon the onset of cancers, appears to promote tumor progression and therapeutic resistance (Gray et al., 1953; Vaupel and Mayer, 2007) The currently accepted hypothesis is that during the formation of a tumor, the vasculature expands in a disorganized and chaotic manner due to the rapid cell division thus resulting in the formation of areas with poor blood flow. This is directly translated into tumor cells present in environments that are deprived of oxygen and nutrients.

**Figure 1:**
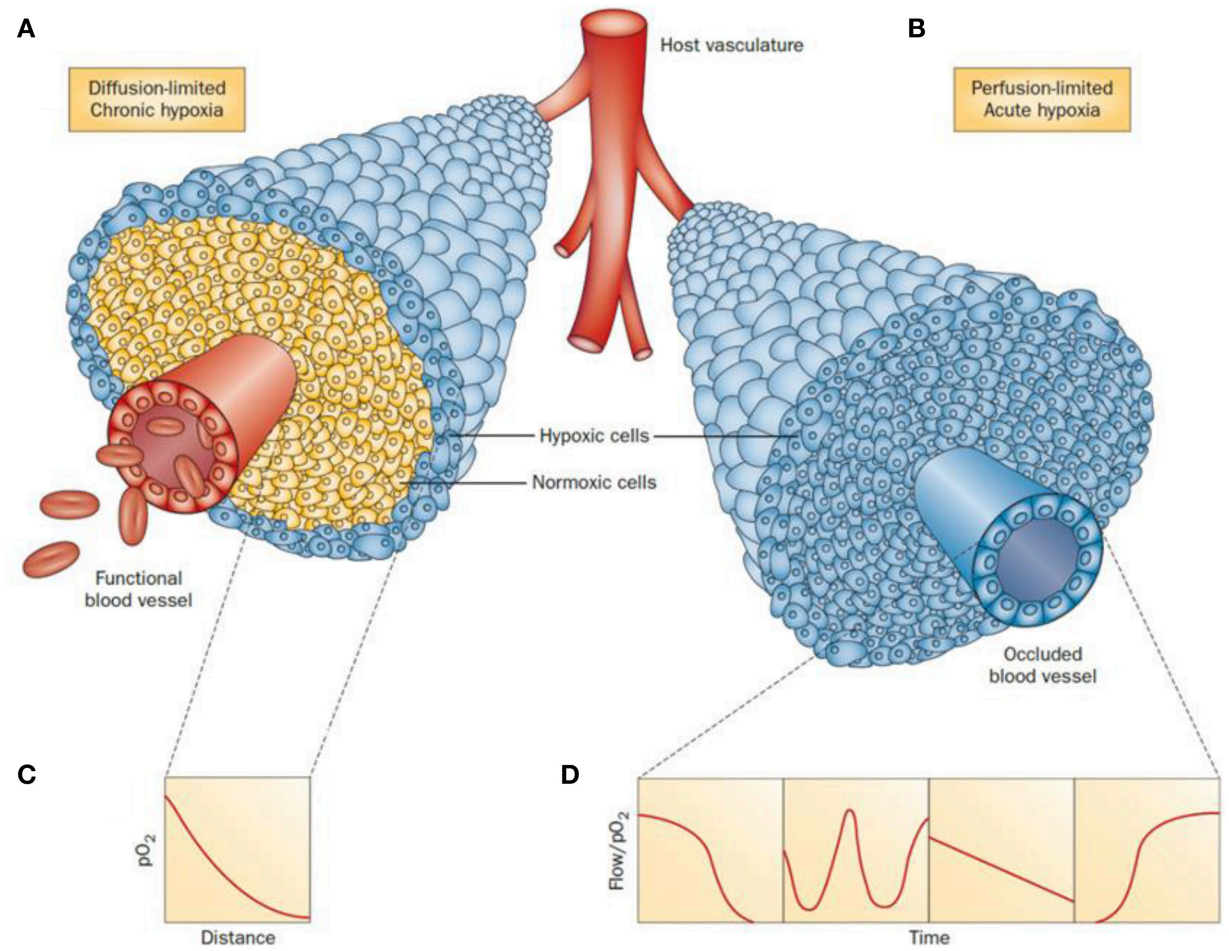
Schematic representation of tumor cells distribution under chronic **(A)** and acute **(B)** hypoxia. In a diffusion-limited chronic hypoxia condition **(A)** the oxygen molecules diffusing from the vessels are used by normoxic cells which result in a decrease of pO_2_ **(C)**. The reduced level of molecular oxygen triggers the proliferation of hypoxic cells at the periphery of the blood vessels. When the vessels are functionally compromised, all the cells around the capillary become acutely hypoxic. Representative flows of pO_2_ over time in the occluded region are shown for comparison **(D)**. Adapted with permission from Horsman et al. (2012).

Tumor progression is regulated by hypoxia-inducible factors (HIF)-1 and -2 (Hahne et al., 2018). (HIF)-1 is a transcription factor that is activated as a result of genetic alterations that stimulate oncogenes and deactivate tumor suppressor genes (Xia et al., 2012) Hypoxia effects are measurable from ca. 100 μm distances from the tumor blood vessels (Vaupel et al., 1989). Broadly, two types or hypoxia can be found depending on the situation and blood supply of the affected cells: chronic or acute hypoxia (Figure 1). In the first case, chronic or diffusion-limited hypoxia occurs as a result of the increased distances of the cells to the microcapillary due to the disorganized vasculature of a tumor. Chronic hypoxia can result in mutations and genetic instability. The second type, acute or perfusion-limited hypoxia, is believed to be caused by the variations and progressive reduction of the flow and transport of molecular oxygen diffusing from the blood vessels to the surrounding cells (Höckel and Vaupel, 2001). In both cases, hypoxia plays an essential role in the metabolic adaptation of tumor cells by activating transcription of target genes used to regulate several biological processes including angiogenesis, cell proliferation and survival, glucose metabolism, pH regulation and migration.

Hypoxic conditions can manifest in biochemical recurrence of cancer after radiotherapy treatment, for example, within only a few years of treatment in the case of prostate tumors (Milosevic et al., 2012). For cancer patients upon diagnosis, the prognosis is strongly related to the hypoxic content of a tumor. The accurate diagnosis of hypoxia is a crucial element toward deciding on the optimal course of treatment. Surprisingly, there are currently no accurate molecular biosensors for pO_2_ gradients to delineate tumor hypoxia in prostate cancers. Sensing hypoxia in tumors is particularly important in the early diagnosis of prostate cancer since the aggressiveness of the cancers and patient's prognosis are directly hypoxia-controlled. Tumors are classed as hypoxic when the level of O_2_ partial pressure is lower than 10 mm Hg.

**Figure 2:**
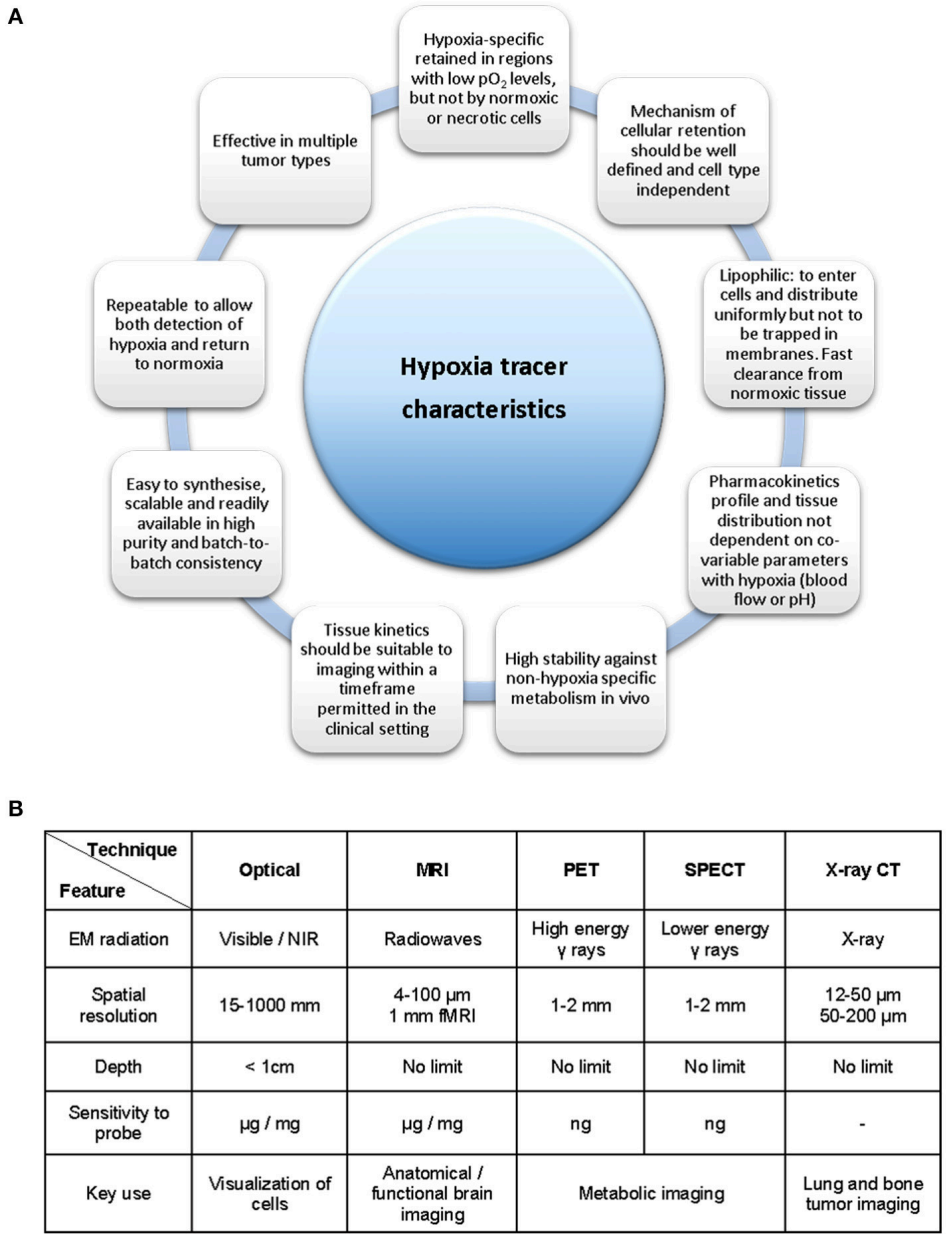
Necessary criteria to be considered in the development of a hypoxia tracer. **(A)** Ideal hypoxia tracer characteristics. **(B)** Available bioimaging modalities and its principal properties and applications. Figure based on data derived (from Fleming et al., 2014, **A** and Long and Wong, 2014, **B**).

The measurement of hypoxia in tumors has been performed directly by oxygen electrodes (Eppendorf probes) or by the use of fiber optic probes (OxyLite) (Griffiths and Robinson, 1999). These methods allow for the direct measurement of the oxygen pressure (pO_2_) in any given area of the affected tissues. However, they also present considerable disadvantages such as being invasive, technically demanding and only providing measurements of accessible tumors (Bussink et al., 2000; Nordsmark et al., 2005). Indirect measurements can also be performed with exogenous (bioreductive nitroimidazoles) or endogenous biomarkers in the form of enzymes such as HIF-1, carbonic anhydrase-IX (CA-IX) or proteins as the vascular endothelial growth factor (VEGF). This approach presents certain advantages such as revealing the micro-regional distribution of hypoxia and are of particular interest in tumor biopsy procedures. These methods are invasive and cannot reliably monitor hypoxia levels over time (i.e., before and after cancer treatment) or in two different regions of the body, without modifying the area through surgery, thus leading to greater patient discomfort. Studies have unequivocally demonstrated that early cancer diagnosis is equally important as the actual cancer treatments toward the survival rate of the patients, which is highly influenced by a timely and precise diagnosis of cancer (Mirabello et al., 2015). Therefore, such timely and precise diagnosis of cancer is paramount toward the ongoing development of sensors, molecular imaging techniques and contrast agents in the attempt to monitor the level and concentration of intracellular and tissue oxygen concentrations and concentration gradients. There is a great need for hypoxia imaging methods capable of measuring and monitoring oxygen that allow for a non-invasive patient's diagnosis, treatment and treatment response. Figure 2 highlights the necessary criteria to be considered when designing a hypoxia tracer. The main characteristics of the ideal hypoxia tracer as acknowledged at the present time are presented in Figure 2A while the available bioimaging techniques are summarized in Figure 2B (Milosevic et al., 2012).

The use of molecular imaging techniques in hypoxia has become a mainstay in the delineation, imaging and progression monitoring of tumors (vide infra, in sections Optical Sensing Approaches: the Role of Oxygen Quenching to Applications of Molecular Oxygen Sensing of Relevance to Cancer Therapy). Molecular imaging has been defined recently as a broad research field concerned with the use of specific imaging agents (or probes) for the use of *in vivo* biological processes at the molecular or cellular level by targeting a specific tissue or cell type (Ametamey et al., 2008).

The techniques used in hypoxia imaging applications comprise PET, single-photon emission computed tomography (SPECT), magnetic resonance imaging (MRI) or optical imaging. To date, PET, SPECT, near infra-red (NIR), luminescence optical imaging and MRI are predominantly applied in pre-clinical research to detect a low level of oxygen *in vivo* (Mees et al., 2009; Lopci et al., 2014). PET is arguably the most current form of research and at the forefront of molecular imaging in clinical practice due to its high specificity and sensitivity. Several reviews have been published in hypoxia imaging, some of them comprehensive such as those focused on the most common hypoxia imaging techniques, nuclear imaging techniques (Mees et al., 2009), PET (Fleming et al., 2014; Lopci et al., 2014), or a combination of PET and MRI (Krohn et al., 2008; Mees et al., 2009; Gaertner et al., 2012; Challapalli et al., 2017; Raccagni et al., 2017). The design and testing of optical imaging probes for oxygen detection have also been reviewed along with *in vivo* experiments focusing specifically on NIR imaging (Liu et al., 2017; Yoshihara et al., 2017; Zhou and Lu, 2017).

The aim of this section is to offer an overview of the available methods focusing on the latest developments in oxygen sensing *in vitro* and *in vivo*. Selected examples have been chosen and analyzed from the viewpoint of their suitability for clinical applications.

## Optical sensing approaches: the role of oxygen quenching

One of the most common methods adopted in optical sensing is based on the quenching of the sensor luminescence by chemical species commonly referred to as quenchers (MacCraith et al., 1993; Tyson et al., 2016; Mao, B. et al., 2017). For a given molecule, M (which acts as the reporting component of the sensor) and a quencher (a given species present in the proximity of M), the luminescence quenching effect (Q) can be described and measured by application of the processes described in Equations (1)–(4) (Demas et al., 1999).

(1) $$\begin{array}{r} \text{M}+\text{h}\nu \to {\text{M}}^{*} \end{array}$$

(2) $$\begin{array}{r} {\text{M}}^{*}\to \text{M}+\text{h}\nu \end{array}$$

(3) $$\begin{array}{r} {\text{M}}^{*}\to \text{M}+\Delta \end{array}$$

(4) $$\begin{array}{r} {\text{M}}^{*}+\text{Q}\to \text{M}+\text{Q} \end{array}$$

The presence of a quencher in the system results in a more rapid depletion of the excited state of the luminescent molecules (M^*^). This typically results in a reduction of luminescence intensity or a shorter emission decay. Changes in luminescence intensity and luminescence lifetime decay can be directly correlated to the quantity of the analyte or quencher (Q) present in the system. Molecular oxygen (O_2_) is well known for being an efficient quencher of fluorescence, due to its unpaired electron system (Ware, 1962). Due to a diffusion-controlled process, the collisions between excited-state fluorescence molecules and O_2_ result in the decay of M^*^ to its ground state without photon emission thus leading to a reduction in luminescence (Lakowicz, 2006). The collisions between the dye (M^*^) and O_2_ (Q) describe a “dynamic quenching” and follows the Stern-Volmer equation for both luminescence intensity (*I*) and lifetime decay (τ):

(5) $$\begin{array}{r} \frac{I_{0}}{I}=1+K_{SV}\left[Q\right] \end{array}$$

(6) $$\begin{array}{r} \frac{\tau_{0}}{\tau }=1+K_{SV}\left[Q\right] \end{array}$$

(7) $$\begin{array}{r} K_{SV}=k_{q}\tau_{0} \end{array}$$

In Equations (5)–(7), *K*_*SV*_ is used to denote the Stern-Volmer quenching constant, *k*_*q*_ for the bimolecular quenching rate constant and [Q] for the the concentration of the quencher (O_2_). From these equations, the presence of molecular O_2_ is shown to affect the luminescence intensity of the dye in addition to shortening its lifetime decay. The Stern-Volmer equation can therefore predict that the intensity of the luminescence (phosphorescence or fluorescence) of the O_2_ probe, decreases as the concentration of O_2_ or its partial pressure (pO_2_) increases. Although the concentration of O_2_ cannot be measured in an absolute manner, changes in the fluorescence intensity and phosphorescence lifetime decay allow monitoring the variation of oxygen levels. Particularly, time-correlated single photon counting (TCSPC) methods and its imaging application (lifetime imaging methods), can be used for the real-time measurement of oxygen level in solutions, as well as in cells and tissues, by calibrating the phosphorescence lifetime of the molecular probes internalized in the cells against the pO_2_ (O'Riordan et al., 2007; Yoshihara et al., 2015).

### ON/OFF luminescence probes

**Figure 3:**
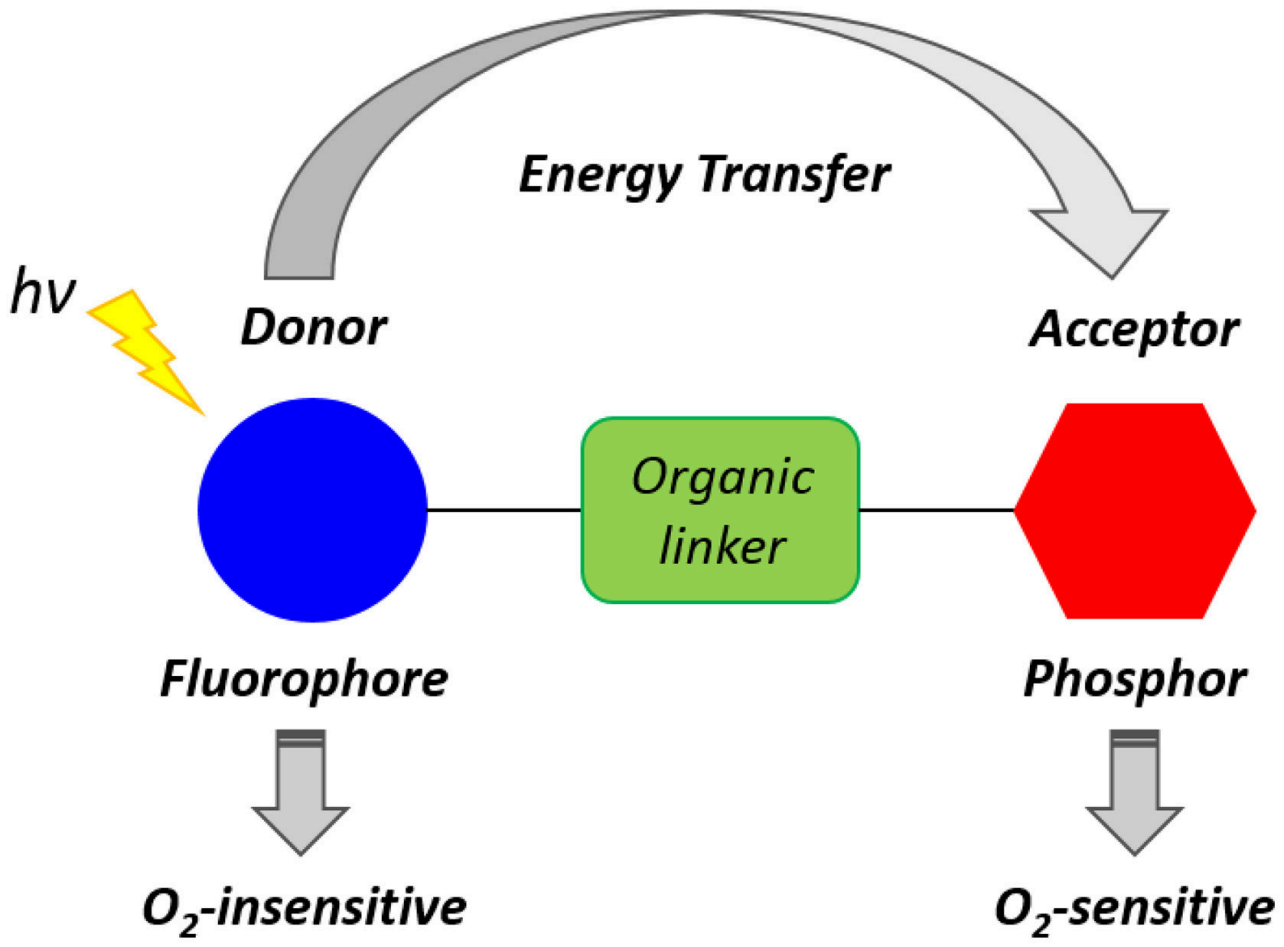
Schematic representation of a generic ratiometric FRET complex oxygen probe inspired by the work of Yoshihara et al. (2012).

The reduced level of intracellular O_2_ that accompanies hypoxia microenvironments results in the accumulation of NADH, FADH_2_, which, in turn, lead to the reduction of O_2_ and formation of ROS. Furthermore, the lack of O_2_ induces anaerobic glycolysis (Gatenby and Gillies, 2004) leading to the increased production of lactic acid and acidosis that is responsible for the acidic extracellular environment, where the measured pH is 6.5–6.9 (Chiche et al., 2010). Xiao and collaborators have demonstrated that hypoxia conditions cause a high level of bioreductase enzymes such as nitroreductase and azoreductase (Cui et al., 2011). Such enzymes are capable of reducing the functional groups of a molecule and triggering an intramolecular rearrangement leading to activated luminescence probes. Several types of luminescent probes incorporating units and functional groups that can be cleaved or modified in the hypoxic environment have been designed. Such luminescent probes have been described as Föster resonance energy transfer (FRET) complexes (Figure 3). These molecules often consist of two units which act as a FRET donor and a FRET acceptor and may be connected by a hypoxia-sensitive cleavable linker. The FRET occurs efficiently when the distance between the donor and the acceptor is within 1–10 nm (Xu et al., 1999). The cleavage of the link results in the disruption of the donor-acceptor resonance energy transfer and subsequent luminescence emission. ON/OFF FRET sensors can, therefore, be used to identify the level of O_2_ and the degree of hypoxia (Cui et al., 2011; Liu et al., 2017).

### Hypoxia-induced chemical modifications of luminescence molecular probes for O_2_

Hypoxic cellular environments can trigger intramolecular modifications that switch and enhance the fluorescence of organic or inorganic molecules (Liu et al., 2017). Hypoxia-sensitive chemical functionalities such as nitroaromatic, quinone, or azobenzene groups have been used to develop hypoxia-sensitive ON/OFF FRET probes. Over the past few years, a variety of hypoxia probes have been designed to incorporate nitro functional groups that can be reduced by nitroreductase enzymes (Elmes, 2016). It is well known that the intracellular reduction of nitroaryl compounds is inhibited by molecular oxygen. Therefore, the qualitative detection of the nitroreduction product can be used to assess the level of intracellular oxygen present. In this work we will focus on the use of transisiton metal complexes as oxygen sensors and probes which have arrived to preclinical or clinical trials. Other complexes/materials as the probes based on difluoroboron β-diketonate poly-lactide acid (PLA) have been previously described elsewhere (Fraser and Zhang, 2009; DeRosa et al., 2015).

**Figure 4:**
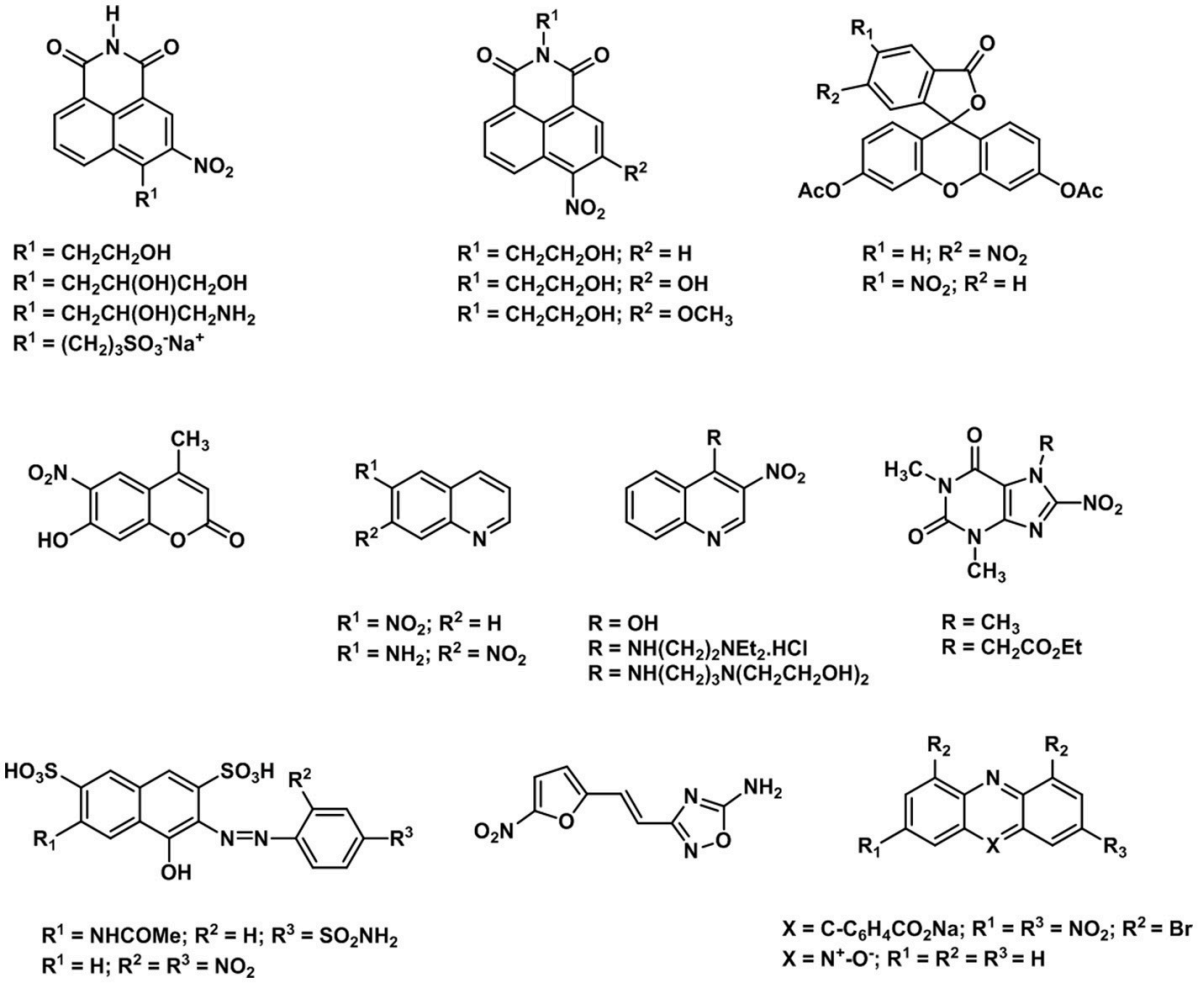
Selected examples of nitroaromatic compounds used as hypoxia molecular sensors.

One of the first examples of nitroaromatic molecules used as molecular hypoxia sensors was reported by Olive and Durand (1983). This study reported the multicell spheroids uptake of a series of nitrofurans which was used to demonstrate that their fluorescence is influenced by the intracellular oxygen concentrations. Such work paved the way for further studies on the cellular metabolisms and applications of nitroaromatic molecules as hypoxic cellular markers (Olive, 1984). A family of hypoxia tracers was developed by Hodgkiss et al. (Begg et al., 1983, 1985; Hodgkiss et al., 1991) in which they reported the synthesis, characterization and cellular metabolism of a series of nitro-aromatic derivatives of N-alkyl naphthalimides. Several of the compounds reported by the authors (Wardman et al., 1984; Hodgkiss et al., 1991) were converted into fluorescent products when incubated within hypoxic mammalian cells. However, the drawback to this was that several naphthalimide compounds were deemed to be DNA intercalators and therefore were not deemed suitable for *in vivo* tests. Other benzene derivatives such as naphthalene acids, 3-nitrophthalimide, coumarin quinoline and fluorescein derivatives, purines, indazole and nitrocaffeine have also been tested by the same authors (Figure 4; Hodgkiss et al., 1991) although none of these compounds showed significant differential fluorescence effects when exposed to hypoxic cellular environments. However, resazurin and nitroacridine contain reducible N-oxide functional groups showed a much more significant fluorescence intensity in hypoxia environments which confirms that nitroaromatic molecules could have been used as an indicator of the selective reduction of the N-oxide function in hypoxic cells (Hodgkiss et al., 1991).

The potential to use the nitrofuran motif for the formation of a selective fluorescence imaging probe and indirect nitroreductase tracer was revised by Ma et al. (Li et al., 2013). This study demonstrated that the fluorescence enhancement associated with the reduction of the nitro group of 7-[(5-nitrofuran-2-yl)methoxy]-3H-phenoxazin-3-one is directly proportional to the concentration of nitroreductase in the range of 15–300 ng/mL and can be detected between 550 and 585 nm by confocal microscopy. As a result, the detection of endogenous nitroreductase in HeLa and A549 cells was achieved with a detection limit of 0.27 ng/mL (Li et al., 2013). The use of such probes *in vivo* experiments is, however, limited by the excitation and emission wavelengths employed in the experiments.

Organic fluorophores usually require excitation wavelengths within the ultraviolet region to generate emissions in the visible region (Hemmer et al., 2016). Radiation at UV wavelengths induces photobleaching of the organic probes thus restricting the usage time, generating tissue autofluorescence and increasing the background emission (Hemmer et al., 2016). Moreover, UV-vis light can only partially penetrate biological tissues and be potentially absorbed by endogenous biomolecules such as hemoglobin (Okuda et al., 2012). The deeper imaging of biological tissues (500 μm to 1–2 cm) requires the use of NIR excitations (around 650–900 nm; Weissleder, 2001). Recently, the use of NIR light to excite or activate fluorescence probes has been one of the most pursued strategies for achieving deep-tissue molecular imaging (He et al., 2010). Nagasawa (Okuda et al., 2012), Tang (Xu et al., 2013) and, more recently, Feng (Li, Y. et al., 2015) explored the use of nitroaromatic units linked to cyanine dyes for the imaging of nitroreductase in tumor cells. Cyanines have high extinction coefficients often exceeding 100,000 Lmol^−1^cm^−1^ and are excellent NIR dyes with strong fluorescence intensities and good photostability (Escobedo et al., 2010; Luo et al., 2011). A recent review summarized a number of fluorescent probes with a focus on organic dyes containing nitro groups as bioreductive agents in the imaging of tumor hypoxia and bacterial monitoring (Elmes, 2016).

**Figure 5:**
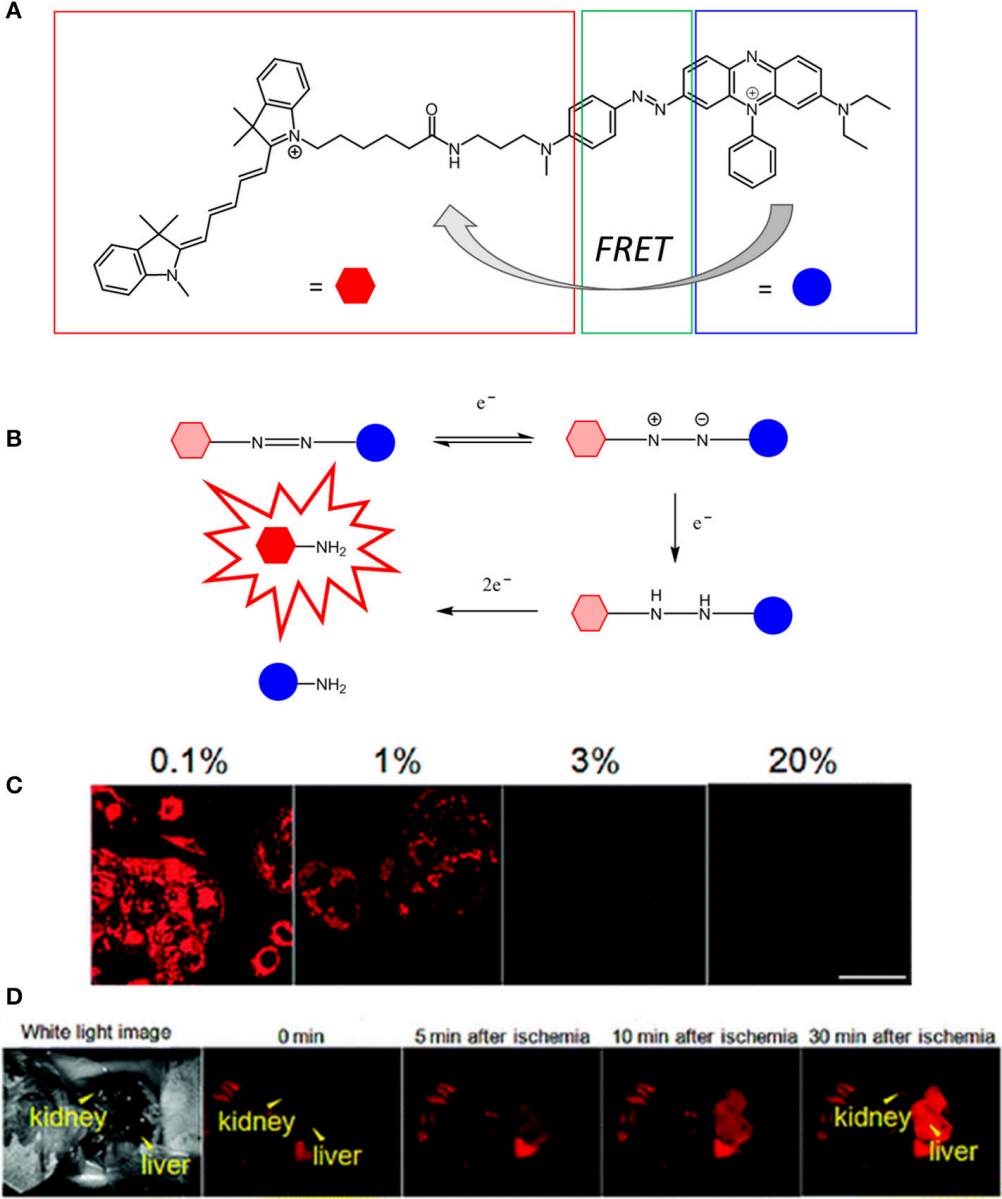
**(A)** Chemical structure of QCy5 designed by Nagato (Kiyose et al., 2010). **(B)** O_2_-dependent stepwise reduction of azobenzene derivatives. **(C)** Confocal scanning fluorescence of MCF-7 cells incubated with 1 μM of QCy5 at various O2 concentrations (0-1–20%). **(D)** Fluorescence images of a living mouse after injection of QCy5 and vessel ligation. Fluorescence was obtained after 30 min (λ_ex_ = 620 nm). Reprinted and adapted with permission from Kiyose et al. (2010).

The first visible-NIR probe for *in vivo* hypoxia imaging was reported in 2010 (Kiyose et al., 2010) by Nagato and collaborators. In this report, they explored a different strategy for pO_2_ sensing by combining a NIR cyanine dye to an azobenzene compound in a bioimaging FRET complex in which the NIR dye and the azo compound act as the donor and acceptor respectively (Figure 5). The authors reported that various hypoxic reductases disrupt the FRET process by reducing the azobenzene units to aniline derivatives. This, in turn, triggers a series of intramolecular rearrangements responsible for the cleavage of the azo bond consequently generating an enhancement of the cyanine dye in the NIR emission. The use of azo structures as hypoxia-sensitive alternatives to nitroaromatic compounds was explored thus allowing for the fluorescence imaging of ischemic organs in live mice. Additionally, Nagano also investigated a new non-FRET-based photo switching concept for the activation of two hypoxia selective rhodamine derivatives, namely MAR and MARS (Piao et al., 2013). In this case, the reduction of the azo bond does not disrupt the FRET interaction between the donor and acceptor units of the molecule but instead it regenerates the original structure of the dyes (2Me rhodamine, Sakabe et al., 2013; and 2Me Si-rhodamine, Kushida et al., 2012).

**Figure 6:**
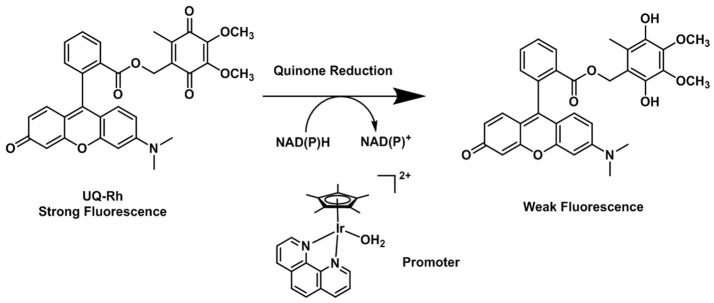
Reduction process of the fluorescent quinone derivative UQ-Rh by NAD(P)H (Komatsu et al., 2014).

The possibility that quinones act as electron acceptor units was demonstrated by Scott et al. (1998). Such species can effectively quench the fluorescence emission of the dye used as the reporting unit. The reduction of the quinone units leads to the formation of the hydroquinones, which are, in comparison, deemed to be electron donor species and unable to quench the emission of the fluorophore (Nohl et al., 1986). By using this strategy, Komatsu and Agira designed a fluorescent ubiquinone-rhodol derivative (UQ-Rh) as a probe for NAD(P)H (Figure 6). The emission of the UQ-Rh was studied in the presence of [(η^5^-C_5_Me_5_)Ir(phen)(H_2_O)]^2+^ (0.5 mM) used as a promoter and a linear correlation between fluorescence intensity and NADPH concentration was determined within a 0.5–5 mM range (Komatsu et al., 2014). The idea of using quinone conjugation to develop new hypoxia-sensitive fluorescence probes was recently extended to ruthenium compounds. By reacting cis-[Ru(bpy)_2_Cl_2_]·2H_2_O with an anthracene quinone derivative of bipyridine, Chao (Zhang et al., 2015) reported the synthesis and characterization of three reversible two-photon luminescent probes for hypoxia. Contrary to what was observed for the FRET complexes of the organic probes reported above, the formation of ruthenium(II) hydroxyanthraquinone is reversible and the hydroxyanthraquinone/quinone (or ON/OFF forms) are in equilibrium with each other. Taking this into account the Ru(II) bipyrydil anthraquinone probes were successfully used to detect repeated hypoxia-reoxygenation cycles *in vivo* in living zebrafish.

Additionally, it has been shown that molecular sensors based on donor-acceptor energy transfer-have been also used to detect a number of intracellular species such as nitroxyl (HNO) (Ali et al., 2017), hydrogen peroxide (H_2_O_2_) (Albers et al., 2006; Wu et al., 2017), H_3_O^+^ (Li et al., 2017), ions such as Hg^2+^ (Singh et al., 2017), Pd^2+^ (Li et al., 2018), Zn^2+^ (Hessels and Merkx, 2015), and mRNA (Ou et al., 2017). Furthermore, several aspects on the use of FRET biosensors for optical signaling of biological processes have been extensively reviewed (Müller et al., 2013; Kaestner et al., 2015; Stumpf and Hoffmann, 2016; Alam et al., 2017; Bohórquez-Hernández et al., 2017; Zheng et al., 2017; Ujlaky-Nagy et al., 2018).

### Metallic complexes as oxygen sensors

The molecular probes described in the section ON/OFF Luminescence Probes, contain redox-sensitive groups which can be used to indirectly monitor different degrees of hypoxia. However, their FRET and optical signals are not directly linked to the concentration of intracellular O_2_ but instead are influenced by hypoxia-induced bioregulations. As a result of this, molecular probes capable of measuring directly the pO_2_ within cells have been developed since the late 80s (Rumsey et al., 1988). Such luminescence probes are usually molecules that are switched off or quenched by O_2_ and can be employed to map the oxygen distribution *in vitro* or *in vivo* (Yoshihara et al., 2017). Over the past few years, at least two strategies have been employed to develop direct and ratiometric O_2_ sensing probes and devices (Liu et al., 2017). The first strategy employs an O_2_-insensitive dye and an O_2_-sensitive chromophore. The latter is also commonly referred to as an O_2_ indicator and undergoes luminescence quenching when in contact with O_2_ molecules.

Thus, ratiometric O_2_ measurements can be obtained by recording and comparing the fluorescence intensities associated with the O_2_-insensitive dye and an O_2_-sensitive probe. Such an approach allows for the correction of the possible effects of the environment (temperature, pH or molecular interactions) on the luminescence emission of the probe.

Ideally, a ratiometric oxygen sensor should satisfy five criteria:

1. Fluorescence and phosphorescence emission maxima are well separated in the spectrum and only the phosphorescence intensity is affected by oxygen quenching.
2. The energy transfer only occurs from the O_2_-sensitive fluorophore to the O_2_-insensitive unit.
3. The intensities of the O_2_-insensitive and O_2_-sensitive dyes are not affected by an intramolecular electron transfer quenching mechanism.
4. The emission of the O_2_-insensitive and O_2_-sensitive dyes are independent of the pH value and polarity of the medium.
5. The probe is efficiently internalized by living cells.

The ratiometric detection of O_2_ can also be achieved by phosphorescence lifetime imaging as previously reported by Nangaku and collaborators (Hirakawa et al., 2015). The author demonstrated that after systematic administration of the Ir(III) complex BTPDM1 [(BTP)_2_Ir(acac), (btp = benzothienylpyridine, acac = acetylacetone)], the phosphorescence probe distributes inside the tubular cells of the mice kidney and its phosphorescence lifetime decay can be directly correlated to the partial pressure of oxygen. Such a phosphorescent system represents a promising intracellular probe to quantify the oxygen concentration *in vivo*. Indeed, the phosphorescence lifetime of the probe can only be affected by the O_2_ concentration and is not influenced by the probe distribution and concentration, or complex intracellular interactions promoted by the intracellular environment. Several different ligand-metal based luminescent probes have recently been employed for O_2_ sensing technologies in living systems and are highlighted below.

#### Porphyrin ligands as scaffolds supporting metallasensors for O_2_

Porphyrins have been extensively studied due to their photochemical properties and significance in the biochemistry of O_2_ as well as in the ferric, Fe(III), and ferrous, Fe(II), ions metabolism (Kafina and Paw, 2017). The four conjugated pyrrole units of the free base porphyrin ligand give rise to the π → π^*^ transitions and result in a strong Soret, or B, band absorbing at ~400 nm and a less intense Q band at ~450–600 nm (Rajora et al., 2017). Chemical modifications to the macrocycle result in Q-bands between 650 and 800 nm.

**Figure 7:**
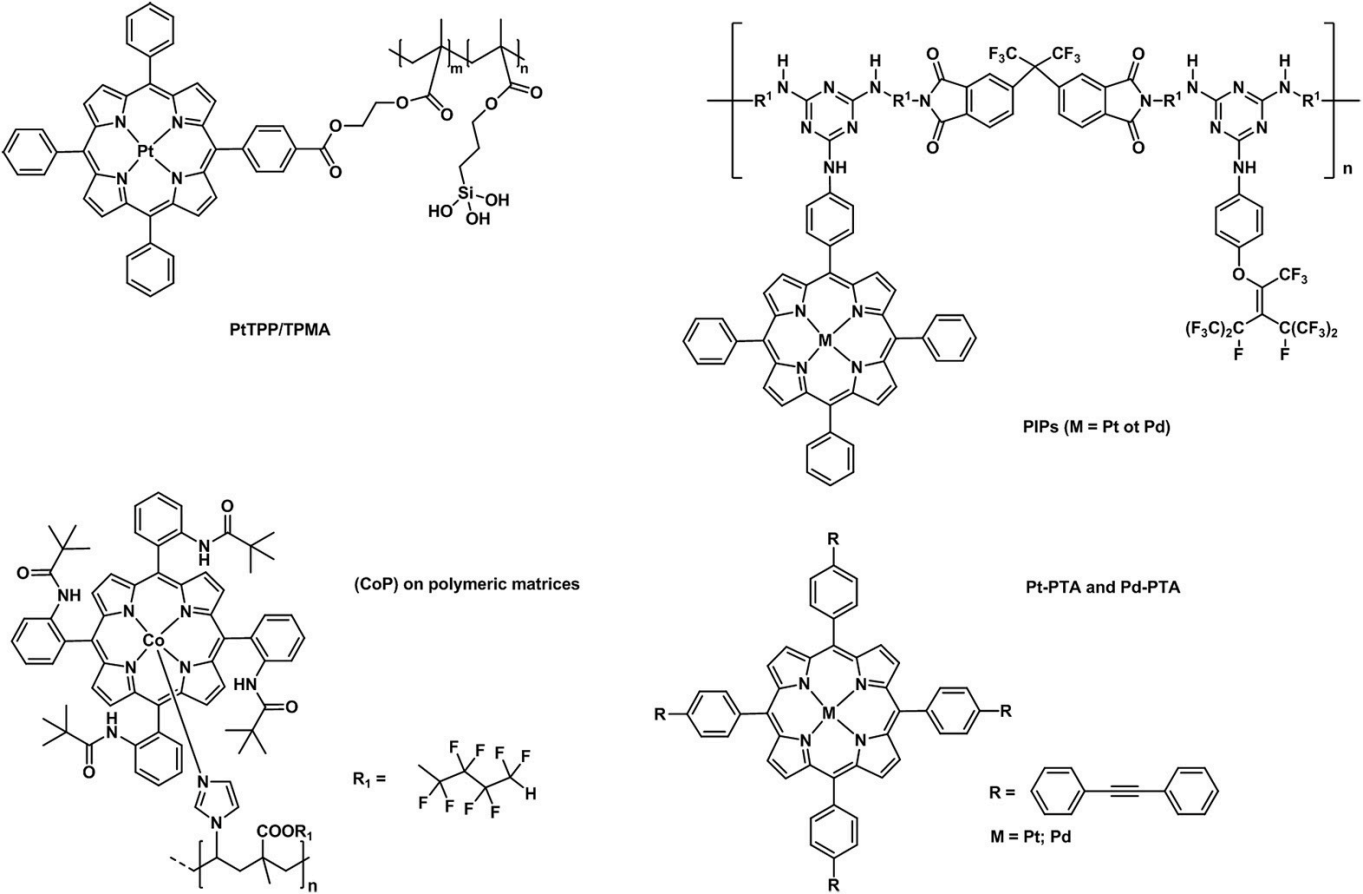
Different examples of metallaporphyrins supported in polymeric matrices (PtTPP/TPMA Mao, Y. et al., 2017, PIPs (Kimura et al., 2017), CoP (Röösli et al., 1997), Pt/Pd-PTA (Önal et al., 2016) applied in the sensing of molecular oxygen.

**Figure 8:**
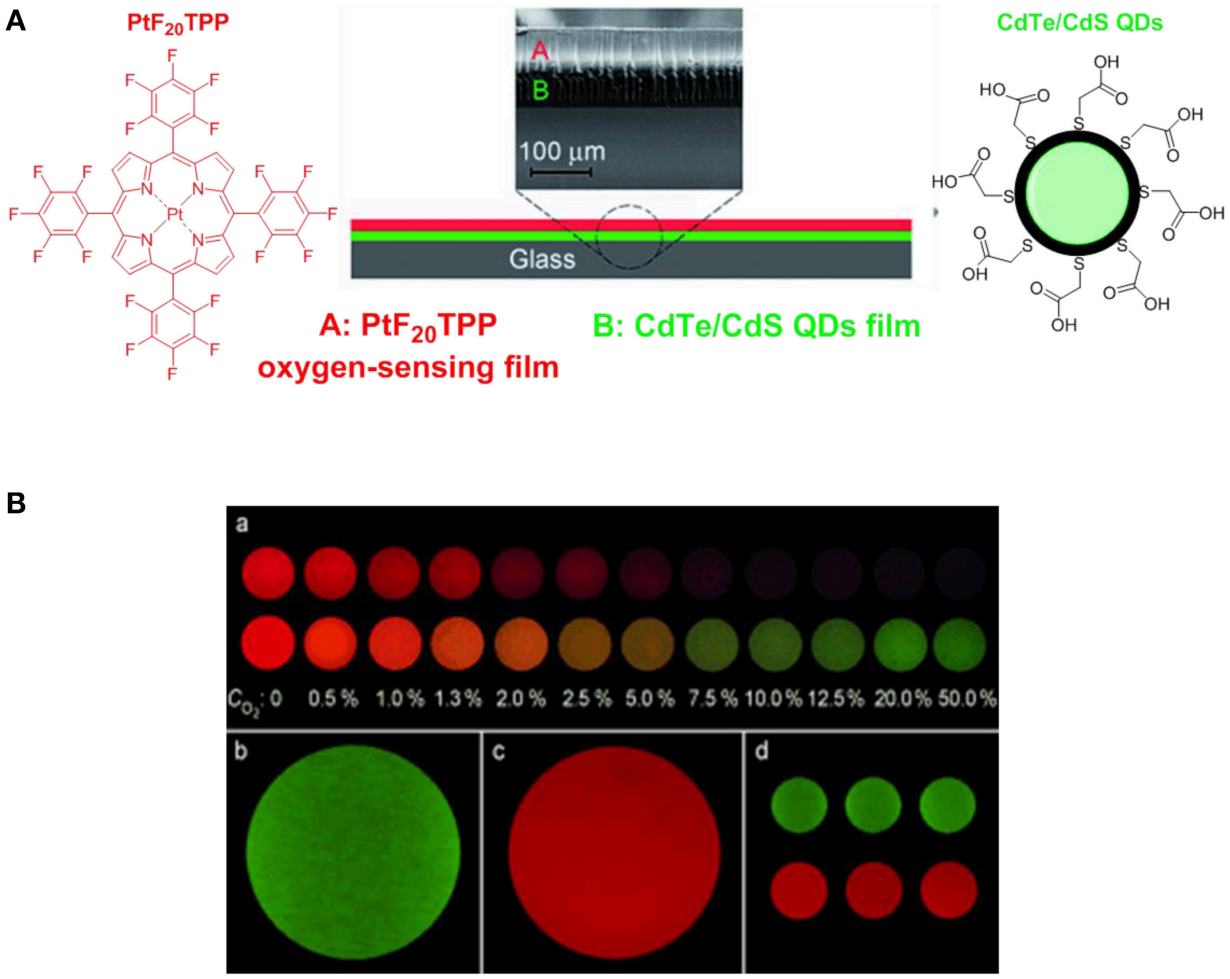
**(A)** Schematic representation of the construction of the [PtF_20_TPP-CdTe] developed by Wang, X. -D. et al. (2008) used for the quantitative and colourimetric determination of molecular oxygen. **(B) (a)** Apparent colors of the sensor at different O_2_ concentration (0–50%) in the absence (top series) and presence (down series) of the CdTe quantum dots acting as internal standards. Green **(b)** and red emissions **(c)** refer to CdTe QDs and PtF_20_TPP respectively. Three sensing films batches prepared at 25.3°C are also presented **(d)**.

Over the past decades, porphyrins have been studied and used to develop photosensitizers in photodynamic therapy (PDT) agents, nanotheranostics, and various contrast agents employed in PET, SPECT and MRI as recently reviewed by Bryden (Bryden and Boyle, 2016; Spagnul et al., 2017) and Zheng (Rajora et al., 2017). Oxygen sensing technologies have recently attracted a great deal of attention because of their usefulness in oceanography (Hasumoto et al., 2006), meteorology (Uchida et al., 2008) and environmental science (Zheng et al., 2008). Tian et al. (Mao, Y. et al., 2017) developed an oxygen probe by covalently bonding a copolymer of platinum porphyrin (PtTPP)/3-(trimethoxysily)propylmethacrylate (PtTPP/TPMA) onto the surface of a polydimethylsiloxane (PDMS) matrices. Such a “crafting reaction” results in a microstructured three-dimensional matrix denoted as 3D PtTPP/TPMA-PDMS-MPAs with a high accessibility for oxygen molecules which is suitable for the characterization of anoxic systems (Figure 7). The presence of Pt-porphyrins allows for a reversible fluorescence response at different O_2_ concentrations reducing the detection limit for dissolved O_2_ in water to 4.7 μmol L^−1^. Recently, the use of copolymers of fluorescent porphyrins has also been discussed by Kimura and collaborators (Kimura et al., 2017). 5-(3-Aminophenyl)-10,15,20-tristolylporphyrinato complexes of Pt (II) and Pd(II) were utilized as building blocks for the formation of polyimide polymers (PIPs) (Figure 7). The luminescence intensity of these PIPs films was quenched by O_2_ and directly correlated to the oxygen concentration between 0 and 100%. The O_2_-dependent optical response of (*meso*-α,α,α,α-tetrakis(*o*-pivalamidophenyl)porphinato)cobalt(II) (CoP) immobilized on polymeric matrices of poly(octylmethacrylate-*co*-1-vinylimidazole) and on poly(2,2,3,3,4,4,5,5-octafluoropentylmethacrylate-*co*-1-vinylimi-dazole) was also studied by Röösli et al. (1997) In this instance, the Co metal center of the porphyrin plays a crucial role. The selective recognition mechanism of the oxygen sensor is based on the reversible formation of the oxo adduct of the CoP membrane that leads to a CoP-oxo membrane along with a variation of the absorption spectra of the sensor. Although at the time of publication, this work represented a valid alternative to the calorimetric measurement of oxygen, it suffered from the absence of an internal standard which would account for the irreversible oxidation of the cobalt centers. A few years later, this particular drawback was overcome by the work of Wang, X. -D. et al. (2008). Optical composite strips of [meso-tetrakis(pentafluorophenyl)-porphyrinato]platinum(II) (PtF_20_TPP) and cadmium telluride (CdTe) quantum dots (QDs) were also developed and used for rapid colorimetric oxygen determination (Figure 8; Wang, X. -D. et al., 2008). The presence of QDs provided an internal fluorescence standard and a better contrast of the luminescence intensity variations of PtF_20_TPP under different concentration of O_2_ with a resolution of 0.5% (Figure 8). Also using PtF_20_TPP but incorporated into a micelle formed by a block copolymer of poly(ε-caprolactone)-block-poly(ethylene glycol) PEG-b-PCL, Su et al. successfully applied the platinum porphyrin to oxygen sensing in aqueous media facilitating its potential application into biological systems (Su et al., 2012). A similar approach was followed by Wolfbeis (Wang et al., 2011) to generate “self-referenced” PEBBLEs of a hydrophobic polystyrene matrix of 500 nm in diameter incorporating two dyes. PtF_20_TPP appears to have been chosen as an O_2_ sensor probe as it matched the red channel of an RGB digital camera, whilst an N-(5-carboxypentyl)-4-piperidino-1,8-naphthalimide butyl ester emitted in the green channel with a fluorescence signal that is not altered by the different concentration of intramolecular oxygen. The RGB PEBBLEs were internalized in the epithelial normal rat kidney (NRK) cells by endocytosis (Mukherjee et al., 1997) thus allowing for *in vivo* imaging. The ratio of the brightness between the red (oxygen probe) and the green (reference dye) channels was calculated for each pixel and provided the opportunity to identify the heterogenous O_2_ supply in distinct intracellular compartments.

More recently (Önal et al., 2016), phenylacetylide bearing palladium(II) or platinum(II) meso-tetraphenylporphyrins (Pd-TPA, Pt-TPA, Pd-TPP, and Pt-TPP) in the forms of nanofibres were employed together with silver nanoparticles (Figure 7). These microhybrid materials showed enhanced quantum yield and higher sensitivity to O_2_ providing a low limit of detection extending to 7.5 ppm. The authors also reported the lifetime based oxygen sensing properties of such phenylacetylide porphyrin nanofibres (Önal et al., 2017). The oxygen partitions (0.86–0.99) indicate an exceptional permeation of O_2_ and the K_SV_ values confirm an extreme sensitivity of the nanofibres to oxygen which can be detected by studying the variation of lifetime decays at different O_2_ concentrations.

Chlorin e_6_ is a porphyrin derivative of interest in PDT capable of generating singlet oxygen. By conjugating chlorin e_6_ to a cell penetrating peptide, Eggleston et al. (Yaghini et al., 2017) reported that such porphyrin-like molecules can efficiently be used in PDT and employed in photo-induced cellular internalization, which is a new technology to increase the intracellular delivery and efficacy of nano-sized biotherapeutics. This work opened the way to the use of porphyrins not only as effective near infrared dyes for bioimaging or therapeutic agents but also as promoters of drug delivery (Yaghini et al., 2017).

#### Iridium-based metal complexes for O_2_ detection in living systems

**Figure 9:**
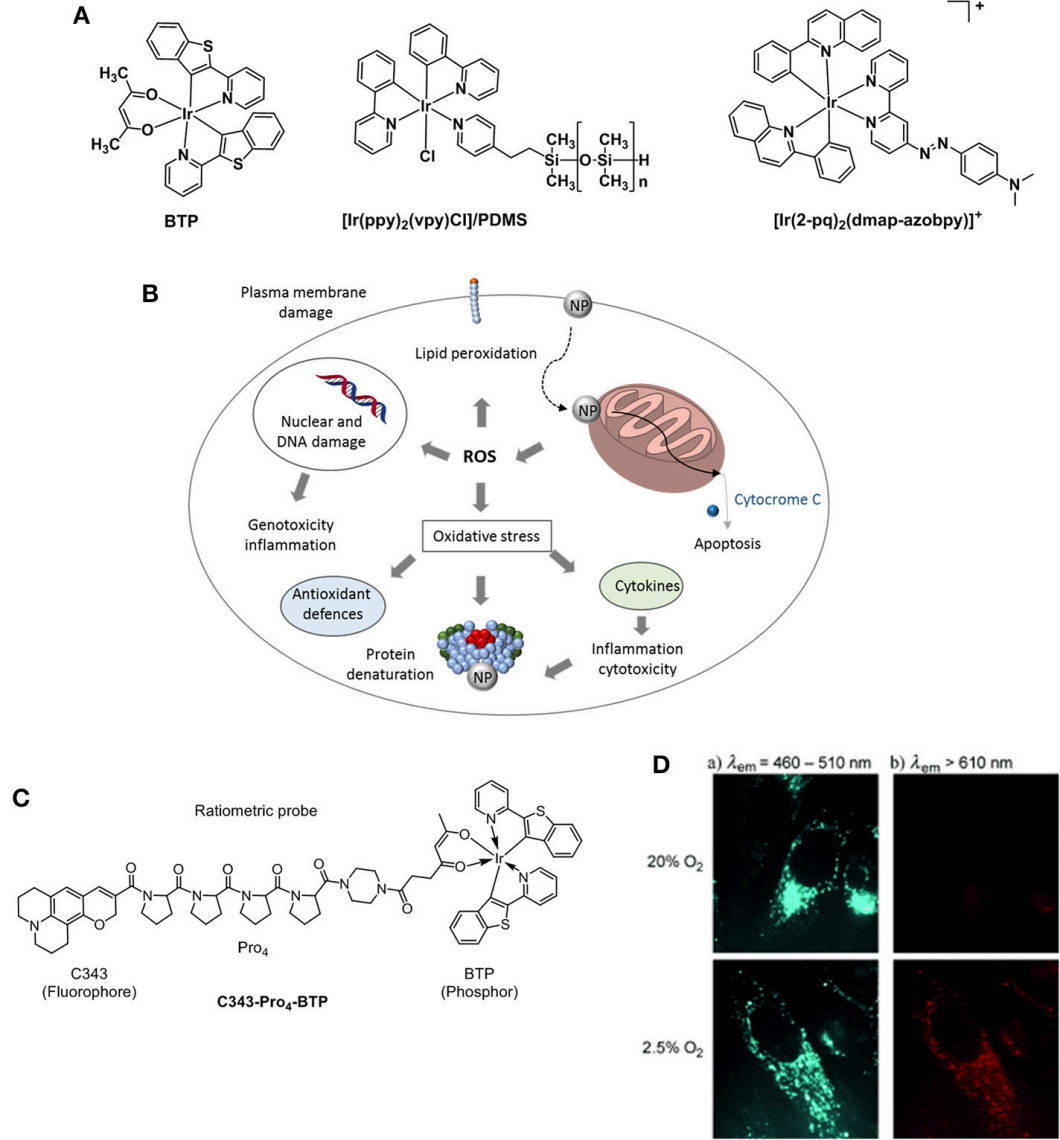
**(A)** Structures of Ir probes [(BTP)_2_Ir(acac)] (Hirakawa et al., 2015), supported probe [Ir(ppy)_2_(vpy)Cl]/PDMS (DeRosa et al., 2003), and [Ir(2-pq)_2_(dmap-azobpy)]^+^ (Sun et al., 2015). **(B)** Mechanisms related to nanoparticle toxicity through production of ROS and oxidative stress. Adapted from Sanvicens and Marco (2008). **(C)** Structure of the ratiometric O_2_ probe C343- Pro_4_-BTP (Yoshihara et al., 2012). **(D)** Luminescence microscopy imaging of HeLa cells incubated with C343- Pro_4_-BTP under 20 and 2.5% of oxygen (λ_ex_ = 400–410 nm).

[Ir(ppy)_3_] (ppy = 2-phenylpyridine) was one of the first iridium complexes examined as a luminescence O_2_ sensor (Vander Donckt et al., 1994). This work has led to the development of further studies on iridium(III) complexes for O_2_ sensing applications proving that the luminescence and in particular, the phosphorescence intensity for this class of compounds is dependably quenched by increasing O_2_ levels (Amao et al., 2001). A non-anionic variation of [Ir(ppy)_3_] was proposed by DeRosa et al in 2003 (DeRosa et al., 2003). The authors reported the synthesis of [Ir(ppy)_2_(vpy)Cl] (vpy = 4-vinylpyridine) and its incorporation in a poly(dimethylsiloxane) (PDMS) matrix which resulted in a longer sensor response time (Figure 9A). Iridium(III)[(2-phenylpyridine)-2-(4,4′-bis(2-(4-N,N-methylhexylaminophenyl)ethyl)-2-2′-bipyridine)]^+^ chloride or N948, was developed in an attempt to create a minimally invasive method to measure the O_2_ concentration within the retina tissue (Ergeneman et al., 2008). At the time, the Ir probe presented many advantages compared to other analogous ruthenium metal complexes ([Ru(bipy)_3_]Cl_2_ or [Ru(phen)_3_]Cl_2_) which include higher quantum yield, higher photostability, longer lifetime and a stronger absorption in the visible spectrum. Subsequently, it was selected and incorporated into a polystyrene matrix highly permeable to O_2_. In turn, the matrix was dissolved in chloroform and the solution was used for dip coating the magnetic spheres of the sensor. Driven by external magnetic fields, the spheres represented a significant improvement in the field of controlled wireless sensor devices for *in vivo* intraocular applications (DeRosa et al., 2003). The immobilization of metallic probes for oxygen sensing on solid supports in the form of nanoparticles is also a promising strategy toward hypoxia therapy as nanoparticles are known to target mitochondria in cells. In presence of the nanoparticles there is a disruption of the mitochondria activity that leads to the production of ROS which triggers a number of responses including oxidative stress, inflammation or membrane damage that ultimately resutls in cytotoxicity and cell death (Figure 9B; Sanvicens and Marco, 2008).

A highly selective phosphorescent iridium-based probe [Ir(2-pq)_2_(dmap-azobpy)]^+^ (2-pq: 2-phenylquinoline; dmap-azobpy: 4-(2,2′-bipyridin-4-yldiazenyl)-N,N-dimethylaniline) was reported by Sun (Sun et al., 2015). The probe was inactive in solution but when treated with rat liver spheroids under hypoxic conditions, a 59-fold enhancement in phosphorescent intensity. Tobita and Zhang (Zhang et al., 2010) reported the synthesis of a red light–emitting bis(2-(2′-benzothienyl)-pyridinato-N,C3′)iridium(acetylacetonate) (BTP) and its applications toward *in vitro* and *in vivo* hypoxia imaging (Zhang et al., 2010). The study proved that the red phosphorescence of the complex was advantageous for tissue penetrance and its lifetime (5.8 μs) encouraging for O_2_-induced quenching. The probe was injected into SCC-7 tumor-bearing mice and the resulting BTP imaging reliably detected in 2 mm sized diameter tumors, where the highest intensity recorded measurements were taken 2 h after injection. An evolution of the BTP probe was proposed by the same group 2 years later where the authors reported an efficient iridium(III) molecular sensor for monitoring the oxygen level in living cells (Yoshihara et al., 2012). The ratiometric probe was designed to have a blue emitting coumarin unit (C343) acting as the O_2_-insensitive fluorophore and the O_2_-sensitive BTP moiety which is functionalized with a carboxylic group capable of reacting with a rigid organic link (Pro_4_) bridging coumarin and BTP (Figure 9C). The ratio between fluorescence and phosphorescence of C343-Pro_4_-BTP has been found to be dependent on the oxygen quantity in HeLa cells (Figure 9D) suggesting the potential for the probe to be used in O_2_ mapping in living cells and tissues. A similar approach was followed by Zheng where a poly(N-vinylpyrrolidone) iridium-(III) complex (Ir-PVP) was prepared and a nanosensor formed by combination with poly(ε-caprolactone)-b-poly(N-vinylpyrrolidone) (PCL-PVP) (Zheng et al., 2015). The authors find out that the nanosensor is hypoxia-activated and able to detect cancer metastasis *in vivo*. The effect of substituents on the luminescence properties of cyclometalated Ir(III) complexes has been independently studied by Liu (Yu et al., 2017) and Yang (Wang et al., 2017). It has been reported that the presence of F atoms on large conjugated aromatic ligands improved the O_2_ sensing performance of their Ir(III) complexes (Yu et al., 2017). DFT calculations suggested that such structural modifications increase the collision probability between the excited electrons of the Ir complexes and O_2_ molecules resulting in a phosphorescence emission efficiently quenched by O_2_. Particularly, the presence of CH_3_ and CF_3_ substituents at the pyridyl unit of the (diphenylphosphoryl)phenylpyridine ligands allows for a higher phosphorescence quantum yield in a range of 54–64% in CH_2_Cl_2_ (Wang et al., 2017).

The vast majority of Ir-based O_2_ sensors reported thus far, have been developed by incorporating and dispersing the iridium molecules or the phosphorescent Ir compounds into porous matrices. In the solid state, small molecule complexes have shown limited oxygen permeability which, in turn, results in a poor optical response toward oxygen (Liu et al., 2014). The formation of polymer-free *self-inclusive* O_2_ sensors based on organometallic molecules was investigated by Mann (Smith and Mann, 2009, 2012). X-ray crystallographic characterization of microcrystalline cationic complexes suggested that the presence of bulky counter anions promoted the formation of void space and channels. Such crystalline packing can facilitate the permeability and exposure of O_2_ molecules across the sensor molecules thus achieving an efficient O_2_ quenching probe involving largely cationic *self-inclusive* O_2_ sensors without the use of a polymeric membrane. Li and Xiao developed *self-inclusive* O_2_ sensors which are able to combine microcrystal thin-films of neutral Ir(III) complexes with relatively high sensitivity toward O_2_ and linear Stern-Volmer behavior (Li M. et al., 2015).

#### Ruthenium-based metal complexes for the O_2_ detection

**Figure 10:**
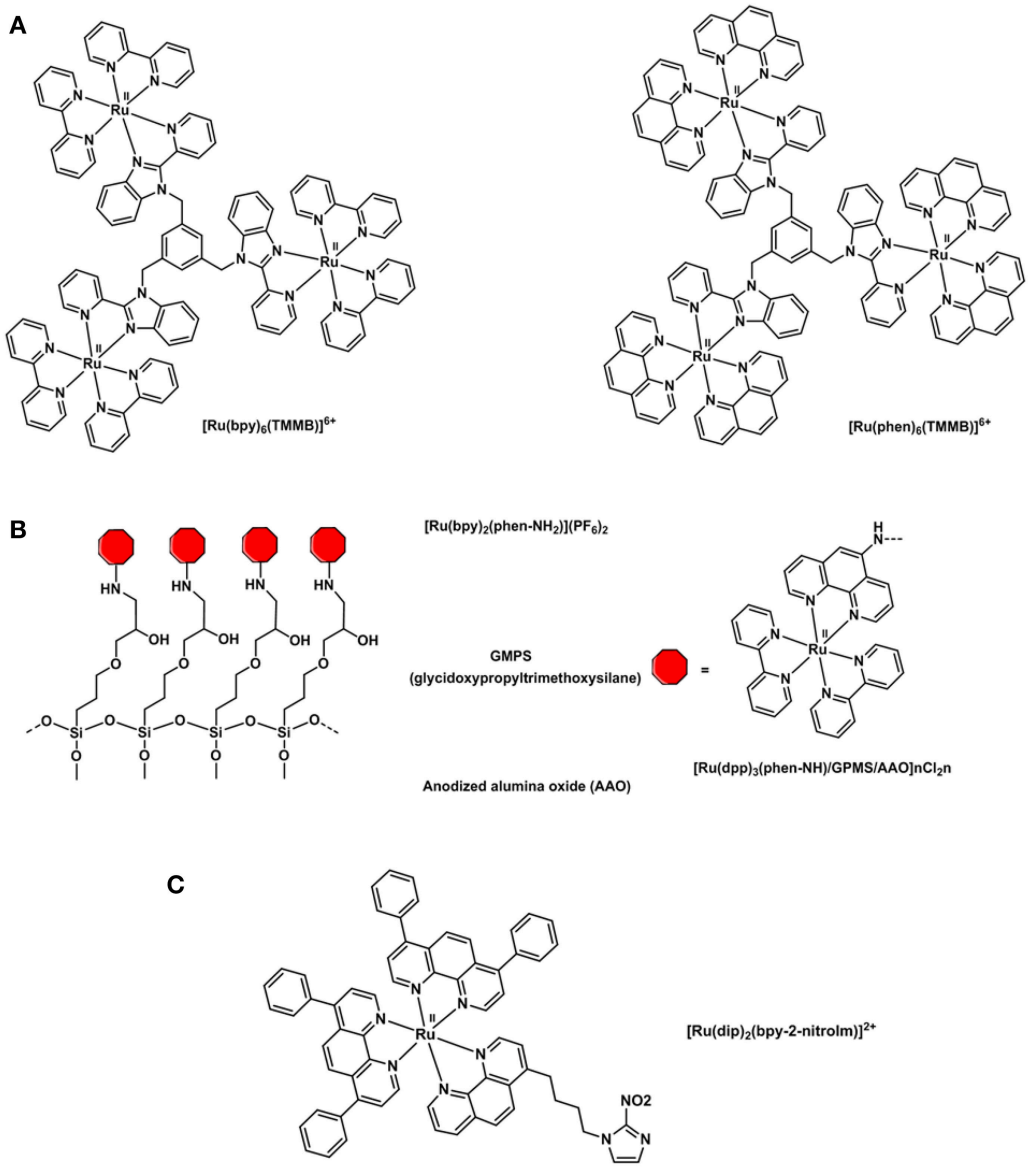
Structural representations of **(A)** oxygen sensitive tripodal TMMB ruthenium complexes (Wang, B. et al., 2008), **(B)** [Ru(bpy)_2_(phen-NH)-] complex incorporated into a solid support for the preparation of an oxygen sensor (Cui et al., 2015) and **(C)** [Ru(dip)_2_(bpy-2-nitrolm)]^2+^ complex (Mazuryk et al., 2014).

Polypyridyl adducts of Ru(II) have been widely studied for O_2_ sensing applications (Amao, 2003). The use of ruthenium tris(2,2′-dipyridyl) dichloride hydrate, [Ru(dpy)_2_Cl_2_]·H_2_O, for quantitative imaging of O_2_ in single cells (J774 macrophages) was first described in 1997 by Gerritsen *et al*. (Gerritsen et al., 1997). The quantitative imaging of oxygen in single cells was studied by fluorescence lifetime imaging. The fluorescence behavior of [Ru(dpy)_2_Cl_2_]·H_2_O revealed that its luminescence intensity is quenched by oxygen and that it is dynamic in solution. Furthermore, the fluorescence lifetime of the compound has been found to be independent of pH, ion concentration and cellular content. The luminescence lifetime of [Ru(dpy)_3_]^3+^ has also been studied and utilized due to its high metal-to-ligand charge transfer, strong UV-vis absorption and large Stokes shift (Bukowski et al., 2005). However, its applicability in intramolecular O_2_ sensing was deemed inferior to that of metalloporphyrins because of its shorter lifetime (<1 μs) and poor cellular uptake efficiency (Yoshihara et al., 2017). The tripodal ligand 1,3,5-tris[2-(2′-pyridyl)benzimidazoyl]methylbenzene) (or TMMB) has been designed by Li (Wang, B. et al., 2008) and coworkers and is used to bind three [Ru(dpy)_3_]^2+^ molecules or three [Ru(phen)_3_]^2+^ (phen = phenanthroline) into a trinuclear starburst ruthenium(II) complex: [Ru_3_(bpy)_6_(TMMB)]^6+^ and [Ru_3_(phen)_6_(TMMB)]^6+^ (Figure 10). The trinuclear Ru(II) molecules were then incorporated into two mesoporous silica matrices: MCM-41 and SBA-15. The resulting hybrid materials were found to be highly fluorescence and their luminescence intensity significantly quenched by oxygen showing good sensitivities and fast response time. Various silica matrices such as MSU-3 (Zhang et al., 2008) and trimethoxysilane (TEOS) xerogel (Roche et al., 2010) were employed in the O_2_ sensing devices in the attempt to improve the thermal and photostability, and visible-light optical transparency. Ru(bpy)_2_(Bpy-Si)Cl_2_ was synthesized by reacting [Ru(dpy)_2_Cl_2_] derivatives with a number of organosilicon precursors (Estella et al., 2010; Lupo et al., 2010; Lledos et al., 2018) resulting in a ruthenium(II) polypiridyl complex bearing a silane NH-(CH_2_)_3_Si(OEt)_3_ pendant moiety that allows for the covalent link between the dye and silica glass films (Malins et al., 1999; Zhang et al., 2008). An alternative to silica sieves and sol-gel matrices was proposed in 2015: anodized alumina oxide was functionalized with the novel polypyridyl Ru(II) complex Ru(dpy)_2_(phen-NH_2_)(PF_6_)_2_ complex (phen-NH_2_ = 5-amine-1,10-phenanthroline) (Cui et al., 2015). The O_2_-sensitive ruthenium compound was covalently anchored to the AAO matrix via a “soft bridge” of 3-glycidopropyltrimethoxysilane (Figure 10). SEM images of the resulting [Ru(bpy)_2_(phen-NH)/GPMS/AAO] hybrid showed an ordered honeycomb structure with uniform pore diameter that is only partially filled with the self-assembled dye. Such a typical structure is responsible for the O_2_ response time which was estimated 100 times faster than any other sol-gel oxygen sensor. The honeycomb structure of the AAO increases the surface area of the system exposed to the O_2_ molecules reducing the time between O_2_ permeation and fluorophore response.

The combination of ruthenium complexes with nitroimidazole groups has also been studied *in vitro* as in the [Ru(dip)_2_(bpy-2-nitrolm)]^2+^ complex displayed in Figure 10C. This complex proved to be selectively retained in hypoxic over normoxic A549 cells and more cytotoxic than cisplatin thus constituting a potential antiproliferative agent in cancer treatment targeting hypoxic tissues (Mazuryk et al., 2014, 2015).

More recently, a nanocomposite material based on Ru(II) molecules and silver nanoparticles (AgNPs) acting as an O_2_ sensor has been developed by Yu (Jiang et al., 2017). The O_2_-sensitive tris(4,7-diphenyl-1,10-phenanthroline) ruthenium(II) dichloride complex, [Ru(dpp)_3_Cl_2_] was encapsulated in polymethyl methacrylate (PMMA) together with the O_2_-insensitive coumarin-6 antenna dye. Finally, the doped PMMA was used to dip coat the AgNPs and tune the luminescence properties of the ruthenium(II). Since coumarin-6 and [Ru(dpp)_3_Cl_2_] have overlapped absorption spectra but significantly different emission profile (^*Ru*^λ_max_ = ~608 nm, ${}^{\text{C}6}\lambda_{\text{max}}$ = ~498 nm) in addition to responding inversely to oxygen, the resulting Ag-doped luminescence fibers were successfully used to measure the photosynthesis and respiratory activities of Chlorella Vulgaris. These results suggested that the system may be used as a ratiometric O_2_ sensor for biological applications in aqueous media.

The immobilization of a tris (1,10-phenanthroline) ruthenium complex in a highly gas-permeable thin silicon-rubber film generated an O_2_-sensing membrane capable of monitoring the *in vivo* pO_2_ distribution on a rat brain surface in an experiment extensively described by Kimura et al. (2007). The system was deemed safe and offered a reliable method to visualize the brain metabolism and pO_2_ mapping without mechanical or chemical invasion (Sanvicens and Marco, 2008).

## Preclinical and clinical imaging of hypoxia

### Positron emission tomography (PET) for hypoxia imaging *in vivo*

PET imaging has been applied in various ways to assist in drug development and to understand drug dosage and treatment strategies (Ametamey et al., 2008). Such studies have provided means to accomplish personalized medical decisions and practices by monitoring individual response to drug delivery. PET imaging agents are radiolabeled with positron-emitting radionuclides, which decay and emit a positively charged particle referred to as a positron that annihilates with an electron after traveling a certain distance in tissue (positron range) generating two gamma rays traveling in opposite directions with a 180° angle. The gamma rays are detected by crystal collimators that produce photons which can be further detected by a photomultiplier tube. The toroidal detector in PET techniques allows for the reconstruction of a three-dimensional image. The PET radioactivity is measured in absolute units (Bq/mL). The radiolabeled probes (“hot” probes) possess the same physicochemical and biochemical properties as the radiolabeled (“cold” probe prototype) compound.

The resolution of PET has improved greatly over the past few years and to date is now within the millimeter order (Pysz et al., 2010; Long and Wong, 2014; Kimoto et al., 2017). Furthermore, the development of multimodal CT/PET systems has substantially improved the accuracy of the imaging process which is now of common use in clinical environments (Cui et al., 2017). Despite the high cost of PET imaging and the special facilities required to undertake work with radioactive compounds this technique has a significant number of advantages such as the ability to image physiological functions and metabolism at the molecular level, a greater sensitivity and the consequent low concentration of probe that is administered to the patient for each scan (Mikhaylova et al., 2017). Furthermore, the variety of available radionuclides makes the chemistry involved in the production of probes incredibly diverse covering isotopic substitution strategies in the short-lived carbon-11 (t_1/2_ = 0.33 h), oxygen-15 (t_1/2_ = 0.033 h), late-stage functionalization for fluorine-18 (t_1/2_ = 1.8 h) compounds and development of chelators for a selection of metals such as copper-64 (t_1/2_ = 12.7 h), gallium-68 (t_1/2_ = 1.1 h) or zirconium-89 (t_1/2_ = 78.41 h) (Long and Wong, 2014). The different energies and half-lives of the positron emitting radioisotopes also allows following different metabolites; the use of longer lived isotopes such as zirconium-89 allows for the metabolism of longer biological processes to be followed like the interaction of antibodies with epitopes (Rousseau et al., 2017). In hypoxia imaging, PET is by far the most popular technique and the technique where most research effort has been devoted due to its high specificity and sensitivity, in addition to being able to detect nanomolar concentration of probes in tissue. However, the current limitation of hypoxia sensing and imaging is that only two PET agents are currently employed in a clinical trial for hypoxia: [^18^F]-fluoromisonidazole ([^18^F]F-FMISO) (Sato et al., 2017; Schwartz et al., 2017) and [^64^Cu] copper-diacetyl-bis(N(4)-methylthiosemicarbazonato) ([^64^Cu][Cu(ATSM)]) (Holland et al., 2007; Hueting et al., 2014).

#### [^18^F]-nitroimidazoles as hypoxia tracers *in vivo*

**Figure 11:**
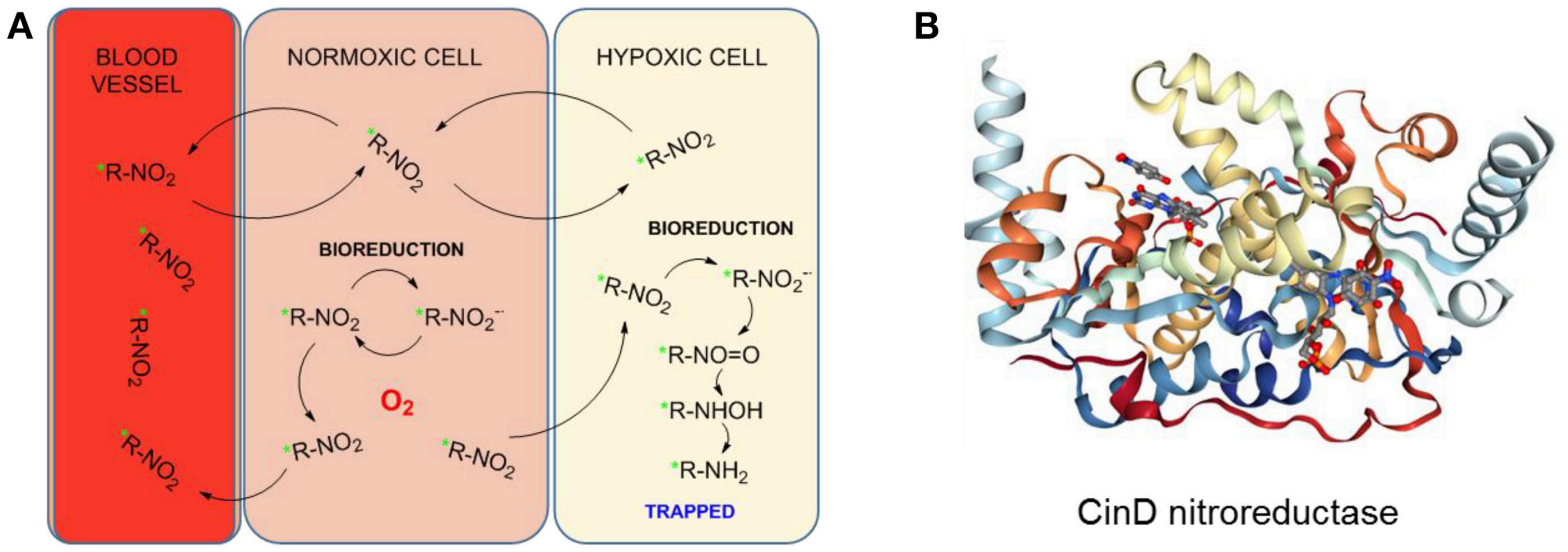
**(A)** Proposed reduction process of a radiolabeled nitroimidazole probe by cellular nitroreductases and subsequent trapping in hypoxic cells. Figured based on data derived from Handley et al. (2011). **(B)** Diagrammatic representation of the secondary structure of the CinD nitroreductase from L. Lactis in complex with nitrophenol and FMN. Image from the RCSB PDB (www.rcsb.org) of Oberholzer et al. (2013).

The metabolism of nitroimidazoles in anaerobic conditions has been known since the 1980s and their hypoxia selectivity originates in the redox properties of the nitro group discussed in the section Hypoxia-Induced Chemical Modifications of Luminescence Molecular Probes for O_2_ (Chapman, 1979; Varghese and Whitmore, 1980). Under normoxic conditions, the nitro group can be reduced in a one-electron process but the process is naturally reverted by re-oxidation with O_2_ molecules in a cycle. However, under hypoxic conditions, the oxygen is negligible and cannot be part of this cycle (Figure 11A). The nitro group can then undergo a 6-electron reduction process in the presence of nitroreductases to the amino derivative. The involvement of functional nitroreductases (e.g., xanthine oxidase) in this process ensures the accumulation of the radiotracer in the viable hypoxic tissue avoiding its accumulation in apoptotic or necrotic cells (Chapman et al., 1981; Rasey et al., 1987). The initial and final species in this reduction cascade are inactive but some of the intermediate species are highly reactive and interact with biomolecules in the cellular environment resulting in the covalent binding of the radiotracer within the hypoxic cellular environments. Therefore, the accumulation of radioisotope in a certain region is an indicator of the presence of hypoxia (Gaertner et al., 2012). A diagrammatic representation of the secondary structure of the CinD nitroreductase enzyme, obtained from its single crystal X-ray structure is shown in Figure 11B (Oberholzer et al., 2013). The enzyme from the Lactococcus lactis bacteria is complexed with the flavin mononucleotide (FMN) and nitrophenol. In this particular case, CinD acts as a copper-induced enzyme that protects the bacteria from oxidative stress originated from nitroaromatic compounds (Mermod et al., 2010).

**Figure 12:**
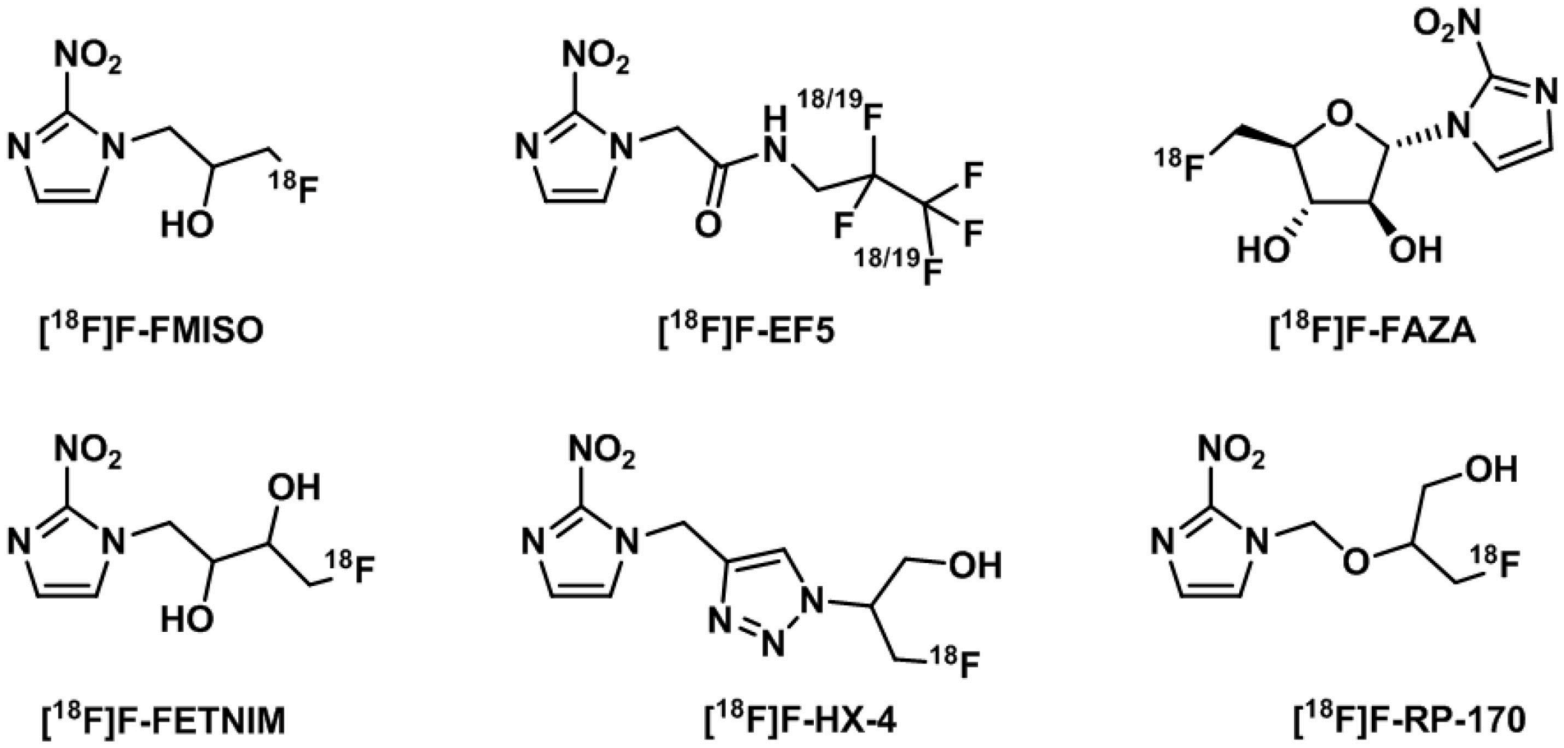
Structures of ^18^F-nitroimidazoles translated into the clinic (Challapalli et al., 2017).

The initial studies in radiosensitizers investigated a number of nitroimidazoles (Figure 12) of which the most applied to date in clinical imaging of hypoxia is [^18^F]-fluoromisonidazole ([^18^F]F-FMISO) (Read et al., 2000). This compound has lipophilic properties which favor cell membrane crossing and diffusion in tissues where its specific retention is observed with reduced oxygen levels (Martin et al., 1990). [^18^F]F-FMISO has shown correlation with hypoxia through accumulation in a number of neoplasms in brain, head and neck, breast, or the lungs. However, one of the main difficulties when designing an imaging study to measure tumor hypoxia is that the same compound can show accumulation within certain types of diseases but not in other which is the case of [^18^F]F-FMISO imaging of pancreatic tumors (Segard et al., 2011).

A comprehensive account on the application of [^18^F]F-FMISO in tracing different tumors has been presented in a number of reviews covering PET imaging, cited in the Introduction (Krohn et al., 2008; Mees et al., 2009; Gaertner et al., 2012; Fleming et al., 2014; Lopci et al., 2014; Challapalli et al., 2017; Raccagni et al., 2017). Despite the general consensus that [^18^F]F-FMISO is the gold standard in clinical research for the measurements of hypoxia, there are also numerous disadvantages associated with its use such as the high non-specific accumulation in normoxic tissue, slow pharmacokinetic profile or formation of metabolites (Challapalli et al., 2017). In particular the first two parameters, influence the clearance rate of the probe from normoxic tissues which at the same time, affects the tumor-to-blood and tumor-to-background ratios rendering images with a moderate or low contrast (Wack et al., 2015). For this reason, much research effort has been applied to the development of new ^18^F-labeled nitroimidazole derivatives with improved lipophilicities to increase their clearance from normoxic tissues and reduce the non-specific retention in normoxic tissues.

[^18^F]-fluoroazomycin-arabinofuranoside ([^18^F]F-FAZA) has been developed as a more hydrophilic version of [^18^F]F-FMISO. The improved hydrophilicity allows for faster clearance from normoxic tissues and a better imaging contrast at earlier time points than FMISO as demonstrated in breast (EMT6) and pancreatic cancer (AR42J) tumors in mice models (Piert et al., 2005). Biodistribution studies revealed differences in humans compared to animals where slower clearance from kidneys in humans was observed although still rendered a favorable radiation risk profile for applications in PET imaging (Savi et al., 2017). Successful clinical studies have been carried out in glioma (Postema et al., 2009), lymphoma (Postema et al., 2009), lung (Postema et al., 2009; Bollineni et al., 2013), head and neck (Grosu et al., 2007) or cervical cancers (Schuetz et al., 2010). In the latter case, an additional imaging technique (CT, MRI) is recommended as [^18^F]F-FAZA is eliminated through the urinary system and a high level of activity remains in the ureters and bladder region interfering with the tumor imaging. Despite the improved properties of [^18^F]F-FAZA with respect to the ones of [^18^F]F-FMISO, the prognostic value of its use has been a matter of debate (Tran et al., 2015). In patients with advanced non-small cell lung cancer, [^18^F]F-FAZA was reported to be a good indicator in the outcome of chemo-radiotherapy compared to [^18^F]F-FDG (Saga et al., 2015). A recent study investigated the hypothesis that imaging patients with lung tumors using [^18^F]F-FAZA, strongly correlated to [^18^F]F-FDG and little additional information about hypoxic regions to direct therapy could be extracted (Di Perri et al., 2017). Pre-treatment [^18^F]F-FDG and [^18^F]F-FAZA PET scans showed that [^18^F]F-FDG PET images display higher tumor-to-background ratio than [^18^F]F-FAZA.

Other ^18^F-nitroimidazoles have been developed in an attempt to improve the properties of [^18^F]F-FMISO. Studies suggested that [^18^F]F-FETNIM is a promising tracer as it presented an improved tumor-to-background ratio compared to [^18^F]F-FMISO for head and neck cancer imaging (Lehti et al., 2004). However, this effect has not been demonstrated for other types of cancer (Wei et al., 2016). Moreover, [^18^F]F-FETNIM production is limited and most of the research has been carried out in Finland (Lehtiö et al., 2001). [^18^F]F-FRP-170 showed improved hypoxic contrast and allowed for a shorter acquisition time as well as being clinically tested in glioma patients (Beppu et al., 2014). The physicochemical properties of nitroimidazoles were also altered to improve their applicability. [^18^F]F-FEF5, a highly hydrophilic probe used in immunohistochemistry, showed greater cell membrane permeability and ability to go into the brain in addition to the reduced tendency to form metabolites. It has been clinically tested in head and neck cancer and glioblastoma with promising results. The main disadvantages of [^18^F]F-EF5 however, are the slow clearance and complex labeling chemistry involving F_2_ gas (Koch et al., 2010). A more hydrophilic nitroimidazole derivative ([^18^F]F-HX4) has been studied in head and neck cancer patients, showing reduced production of secondary metabolites although the tumor-to-reference values were comparable to the ones of [^18^F]F-FMISO. There are indications, however, that this value may improve at later time periods post-injection (Zegers et al., 2013).

#### The hypoxia tracer [Cu(ATSM)] and its radiolabeled analogs: considerations on its mode of action under pO_2_ gradients

[Cu(ATSM)] [copper (diacetyl-bis(N4-methylthiosemicarbazone))] is a well-studied compound of the copper bis(thiosemicarbazonato) complexes family that has been explored as hypoxic markers and radiolabeled with different radioisotopes of copper (Cortezon-Tamarit et al., 2016). [^62^Cu][Cu(ATSM)] was first described to be hypoxia selective by Fujibayashi in the 1990s in ischemic heart murine models (Fujibayashi et al., 1997). Over the course of the years, several studies suggested that [^64^Cu][Cu(ATSM)] possesses improved properties with respect to [^18^F]F-FMISO (Lewis et al., 1999), along with the ability to cross the membrane of hypoxic cells and accumulate in tissues with a low level of O_2_ (Lewis et al., 2001). The structural variations of the thiosemicarbazone backbones were designed to rationalize and improve its cellular uptake and dependency on lipophilicity and redox potential (Dearling and Blower, 1998; Dearling et al., 1998). Although copper-64 (t_1/2_ = 12.7 h) is the most commonly used copper isotope in research clinical practice, other copper isotopes have been used in thiosemicarbazone chemistry such as copper-60 (t_1/2_ = 20 min), copper-61 (t_1/2_ = 3.4 h) or copper-62 (t_1/2_ = 9.74 min) (Blower et al., 1996).

**Figure 13:**
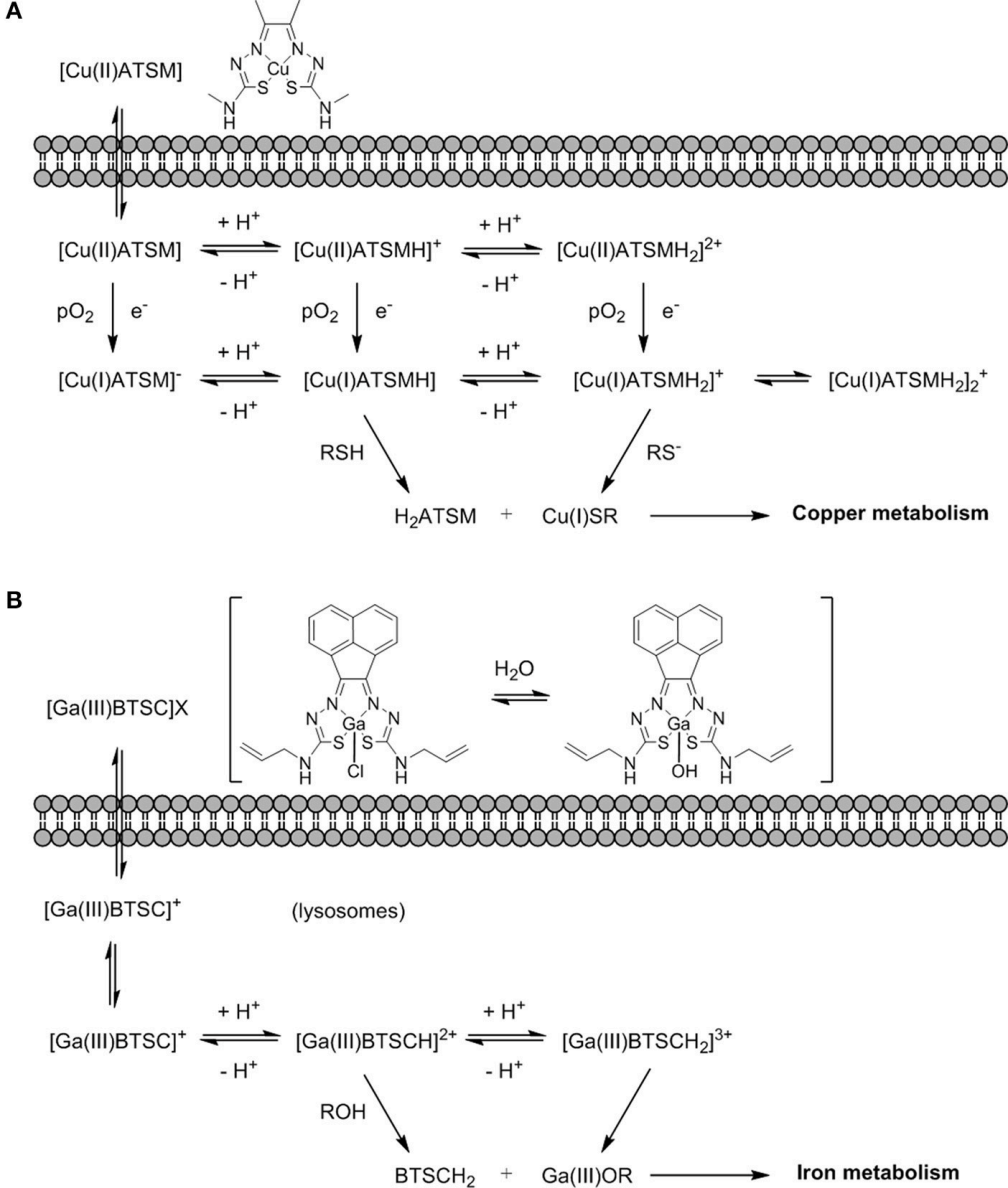
**(A)** An earlier proposing mechanism showing the fate Cu(II)ATSM in a hypoxic cellular environment, involving reduction and decomplexation under reduced pO_2_. Adapted from Holland et al. (2008) and Dilworth and Hueting (2012). **(B)** Proposed non-redox mechanism by analogy with (A) exploring possible fate of [Ga(III)BTSC]Cl in a hypoxic cellular environment involving a simplified view on protonation equilibria and decomplexation (X: Cl^−^, OH^−^) (Alam et al., 2016).

The mechanism of action of [^64^Cu][Cu(ATSM)] has been the subject of intense debate and several spectroscopic and electrochemistry investigations, as well as computational modeling of the systems, have been carried out (Dearling et al., 2002; Maurer et al., 2002; Castle et al., 2003; Holland et al., 2006). The most accepted mechanism now involves the uptake of the Cu(II) complex into the hypoxic cellular environment which can then follow a number of different pathways of reduction and protonation depending on the acidity and pO_2_ that coexist in a subtle equilibrium (Figure 13A). These species can ultimately be transformed into unstable Cu(I) protonated species that dissociate into the protonated ligand and Cu(I) that is sequestered by copper-dependent proteins and introduced into the copper metabolic cycles inside the cell (Holland et al., 2008, 2009) and its mechanism of action appeared to be closely related to that of ^64^Cu-acetate in cells (Dilworth and Hueting, 2012).

The latest contributions to clarify this pathway *in vitro* were carried out by tagging an organic fluorophore to [Cu(ATSM)] and studying the fluorescence lifetime of the complex. In this case, [Cu(ATSM)] proved to be more stable *in vitro* than any other member of the family (Waghorn et al., 2013). Equally, the latest findings *in vivo* appear to indicate that the [^64^Cu][Cu(ATSM)] complex mimics the response of [^64^Cu][Cu(OAc)_2_] thus revealing that copper metabolism plays an important role in [^64^Cu][Cu(ATSM)] imaging displaying processes of copper rather than hypoxia selectivity especially at shorter times (Burdett et al., 1978; Hueting et al., 2014). Consequently, the labeled ATSM probes can be considered as delivery methods for the radioactive metal ions. [^64^Cu][Cu(ATSM)] demonstrated to overcome some of the difficulties presented by [^18^F]F-FMISO, it shows a more rapid uptake, a quicker clearance from normoxic tissues and a greater hypoxic-to-normoxic ratio (Lewis et al., 1999, 2001, 2002). For this reason, radiolabeled [Cu(ATSM)] was tested in clinical trials and has since been demonstrated to provide valuable information for patients with lung (Takahashi et al., 2000; Dehdashti et al., 2003b), cervical (Dehdashti et al., 2003a) or rectal cancers (Dietz et al., 2008).

In addition, [^64^Cu][Cu(ATSM)] and some other members of the copper bis(thiosemicarbazonato) family have shown promising results in the imaging of cardiac hypoxia with PET although the hypoxia selectivity is produced under smaller pO_2_ values than the general observations within cardiac hypoxia (Handley et al., 2011).

#### ^68^Ga-labeled compounds as alternative tracers for imaging hypoxia

**Figure 14:**
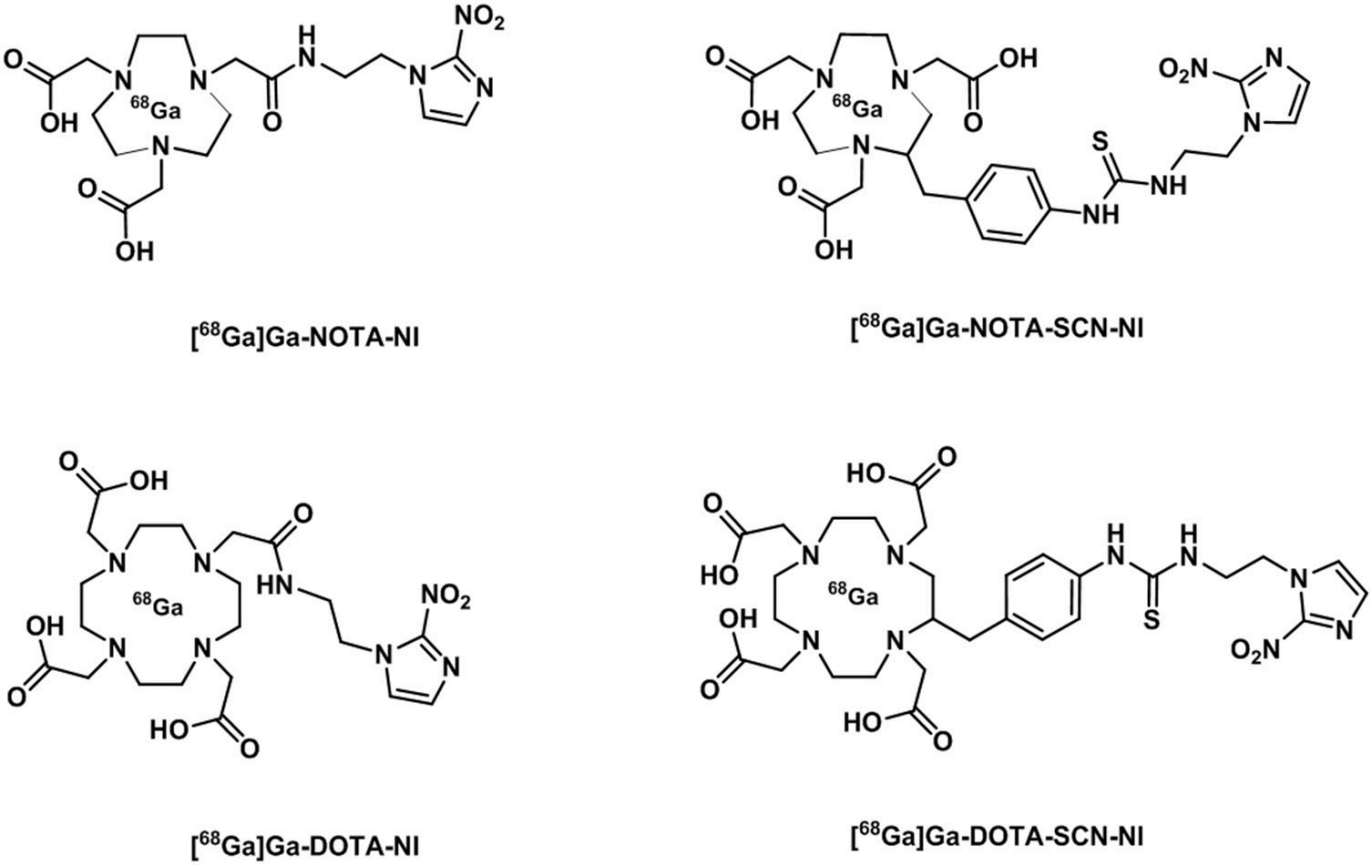
Diagrammatic representation of structures of [^68^Ga]Ga NOTA and DOTA radiotracer bearing nitroimidazole units (Seelam et al., 2015).

The interest in gallium-68 as a radionuclide has been propelled due to its lifetime of ~68 min that albeit shorter than that of fluorine-18 (109 min) is still adequate to allow for chemical transformations to take place and labeling of numerous compounds. Moreover, gallium-68 can be produced in a generator from the long life isotope germanium-68 avoiding the need of a costly synchrotron or access to nuclear reactors (Velikyan, 2014). Gallium-68 labeling has also been applied to probes containing a nitroimidazole unit and a macrocyclic chelator such as 1,4,7,10-tetraazacyclododecane-1,4,7,10-tetraacetic acid or 1,4,7-triazacyclononane-1,4-diacetic acid, commonly referred to as DOTA or NOTA respectively (Figure 14) which are both able to complex gallium-68. In the recent past, novel ligands such as N,N'-bis[2-hydroxy-5-(carboxyethyl)benzyl]ethylenediamine-N,N'-diacetic acid (HBED-CC) have been applied to the complexation of gallium-68 as the metalation is considerably faster than with DOTA at room temperature. HBED-CC has been successfully applied in the theranostics of prostate cancer when conjugated to the prostate-specific membrane antigen (PSMA) (Virgolini et al., 2017) but the combination with oxygen sensitive groups is yet to been investigated. Gallium-68 labeled nitroimidazole complexes were studied in mice models with induced (xenografted) tumors for CT26 (colon cancer) (Seelam et al., 2015), A549 (Wu et al., 2015) or 3LL (Fernández et al., 2013) (lung cancer cell lines). The comparison with [^18^F]F-FMISO revealed successful hypoxia imaging and improved clearance properties. Recent examples have appeared in the literature and showed an affinity for the (HIF)-1 regulated carbonic anhydrase-IX receptor (CA-IX), a well-known endogenous marker for hypoxia (Supuran, 2017). Two similar systems involving benzenesulfonamides to target CA-IX and macrocyclic chelators to complex gallium-68 were reported and evaluated in murine models. Biodistribution studies showed that the probes accumulated preferentially in hypoxic tissue with low blood uptake (Lau et al., 2016; Sneddon et al., 2016). An aromatic thiosemicarbazonato derivative, structurally related to [^64^Cu][Cu(ATSM)], was found to be kinetically stable and hypoxia selective in EMT6 cells when labeled with gallium-68. The probe showed the potential to enhance the stability of the thiosemicarbazonato complex compared to the ATSM backbone and opened up new opportunities for the radioisotope to be used with this ligand family (Alam et al., 2016). The potential hypoxia selectivity mechanism of the complex was suggested to be related to monitoring of iron metabolism changes (altered under reduced pO_2_) as a result of protonation equilibria, decomplexation, and sequestration of gallium in physiological media by iron chelators (Figure 13B; Peyssonnaux et al., 2008; Torti and Torti, 2013).

### Single-photon emission computed tomography (SPECT) and relevant radiotracers for hypoxia imaging *in vivo*

SPECT is also capable of imaging the entire body (Papagiannopoulou and Hadjipavlou-Litina, 2018). In comparison to PET, SPECT makes use of a radionuclide that decays emitting γ rays. The rotating detector provides information on the angle of incidence of the gamma rays. The radioisotopes used in SPECT have, in general, longer half-lives such as that for technetium-99m (t_1/2_ = 6 h) which is the most popular choice of radioisotope as it can be generator produced by the parent ion, molybdenum-99 (Long and Wong, 2014). Radioisotopes of iodine (iodine-123, iodine-125) have been used to label nitroimidazole derivatives as iodoazomycin arabinoside (IAZA) that was developed in the 1990s and showed promising accumulation in EMT6 tumors. However, the clearance rate from the tissue was slow and comparable to the initial nitroimidazoles applied in fluorine-18 imaging. The IAZA tracer also presented stability concerns and images obtained at short times appeared to correspond to free radioiodide rather than the intact compound (Mannan et al., 1991).

One of the first technetium-99m tracers clinically evaluated was [^99m^Tc]Tc-BRU59-21 which showed promising results *in vivo* with greater stability and reduced production of metabolites of earlier [^99m^Tc]Tc-nitroimidazole tracers (Melo et al., 2000). In head and neck cancer patients, the tracer was found to be safe for clinical use and presented selective accumulation as confirmed by pimonidazole immunohistochemical staining of excised tumors (Hoebers et al., 2002).

One of the best studied SPECT tracers for hypoxia is [^99m^Tc]Tc-HL-91, a simple hydroxymate/oxime chelator that does not contain a nitroimidazole moiety. [^99m^Tc]Tc-HL-91 showed selective accumulation and good correlation with Eppendorf pO_2_ measurements in early *in vitro* and *in vivo* studies (Honess et al., 1998; Zhang et al., 1998). The mechanism of action of [^99m^Tc]Tc-HL-91 remains to be fully understood to date: it is believed to be related to the reduction of the metal. This was supported by comparison to the carbon-14 labeled ligand ([^14^C]HL-91) and technetium-99m complex ([^99m^Tc]HL-91) where the ligand did not show any preferential accumulation (Honess et al., 1998). The selective accumulation was compared to the response to radiotherapy but the results were not conclusive to extract a direct correlation of the use of [^99m^Tc]Tc-HL-91 as a prognostic factor (Suzuki et al., 2003). Clinical studies have been performed in small numbers and direct comparisons with [^18^F]F-FDG carried out, showing a correlation in patients with a number of tumors (Cook et al., 1998). Other studies in patients with recurrent squamous cell carcinoma of the head and neck showed promising outcomes, although the tracer binding did not occur in all cases (Van de Wiele et al., 2001). In the case of patients with non-small cell lung cancer, the imaging before therapy was suggested as an indicator of therapy response and patient survival (Li et al., 2006).

In summary, despite the superior availability of SPECT scanners and the easier availability of some γ-emitting isotopes such as technetium-99m, that is widely available in clinical settings as generator-produced from the parent isotope molybdenum-99, the enhanced spatial resolution and more accurate quantification of the images have enabled PET to become the preferred technique for the imaging of hypoxia in tumors or cardiac ischemia.

### Magnetic resonance imaging (MRI) techniques for pO_2_ imaging *in vivo*

MRI relies on the different relaxation times of the protons in water due to different physiological environments when subjected to a magnetic field. These differences along with the changes in the magnetic field in specific gradient profiles allow for the reconstruction of a three-dimensional image of the patient's body. MRI is characterized by a high spatial resolution (Figure 2B). Certain paramagnetic compounds, affecting the longitudinal relaxation (T_1_) or transverse relaxation time (T_2_), are applied to MRI imaging to improve the contrast of the images obtained (Long and Wong, 2014). The application of MRI in hypoxia is desirable as it does not involve radioactive isotopes and the scanners are now widely available. Furthermore, MRI is able to measure changes in oxygen blood levels directly as oxygen is paramagnetic and shortens spin-lattice relaxation times (T_1_). T_1_ is affected by many other factors occurring *in vitro* and *in vivo* experiments, and acquiring quantitative results is challenging. Blood oxygen level dependent (BOLD) MRI, on the other hand, is able to measure oxygen differences directly by studying changes between hemoglobin (O_2_Hb) and paramagnetic deoxyhemoglobin (dHb) which increases the transverse relaxation time (${\text{T}}_{2}^{*}$) of the water in the circulatory system (Nield et al., 2005). BOLD MRI gives a qualitative measure of pO_2_ in real time and has been applied to brain and tumor imaging (Baudelet and Gallez, 2002).

In dynamic contrast-enhanced (DCE) MRI, a molecular probe (i.e., a chemical compound) is administered to the patient to alter the relaxation times in tissue thus providing an improved contrast. DCE-MRI has been specifically applied to the detection of hypoxia in various tumors in an attempt to correlate the results with the pO_2_ levels in tissue. This goal has been achieved with unequal results in different types of cancers and a direct correlation between Eppendorf probe results (Cooper et al., 2000) and pimoinidazole staining (Hauge et al., 2017) has been observed in cervical cancer or prostate cancer (Hoskin et al., 2007). However, this correlation with pimoinidazole staining has not been fully clarified thus far in other cases, such as at HNSCC (Newbold et al., 2009; Donaldson et al., 2011) or [^18^F]-FMISO uptake in glioblastomas (Swanson et al., 2009).

Magnetic resonance spectroscopy (MRS) takes advantage of the MRI setup to acquire ^1^H, ^31^P or ^19^F spectra of metabolites *in vivo*. Using this technique, certain metabolites known to be overexpressed in tumors such as choline or lactate can be followed and their concentration compared to adjacent tissues (Griffin and Shockcor, 2004). The use of ^19^F-nitroimidazoles was considered using MRS as hypoxia-selective tracers where some derivatives were proposed and clinical trials were reported (Seddon et al., 2003; Lee et al., 2009) however, patient cohorts were small and no validation through comparison to other selective probes such as [^18^F]F-FMISO or pimoinidazole staining were carried out.

Interestingly, perfluorocarbons (PFCs) are another family of fluorine-containing compounds relevant to hypoxia imaging that can be observed by ^19^F-MRS. These compounds can have respiratory gas transport potential as they can dissolve large amounts of O_2_ or CO_2_ and it has been observed that T_2_ of some PFCs is closely related to oxygen tension (Gaertner et al., 2012). These combined factors amongst other positive properties of the ^19^F nuclei (spin 1/2, 100% abundance, high sensitivity, no background *in vivo*) make PFCs promising compounds for measuring pO_2_ levels. Several derivatives have been commercialized as blood substituents (e.g., Fluosol®, Oxygent®, Oxycyte®) and can be exploited to measure oxygen levels when incorporated into solid supports or injected directly into the tumor (Nöth et al., 2004).

## Applications of molecular oxygen sensing of relevance to cancer therapy monitoring

The presence of hypoxia is a characteristic feature of solid tumors and has been identified in many neoplasms and related to changes in gene expression and genetic instability as a result of its resistance to apoptosis and decreased DNA repair. It favors the survival of malignant cells in a hostile environment and the expression of an aggressive phenotype that can increase the risk of tumor metastasis (Bristow and Hill, 2008). Furthermore, cancer stem cells have been observed to accumulate and perpetuate in areas with reduced pO_2_ levels and a HIF-related regulatory activity (Li et al., 2009; Tafani et al., 2011). In addition, hypoxia is the cause of resistance to radiotherapy. The reduced presence of oxygen decreases the free formation of radicals which radiotherapy relies on to cause DNA damage to tumor cells (Vaupel and Mayer, 2007). Hypoxic tumors also present chemotherapeutic resistance due to the reduced drug penetration (caused by the irregular vascularization), extracellular acidification, and the aforementioned genomic instability and resistance to apoptosis. As a result, the presence of hypoxia has been used as a negative prognostic factor in a number of diseases, especially cancers (Jubb et al., 2010). The heterogeneity of cancer and the lack of a universal hypoxia detection tracer/technique presents a challenge for the correlation of hypoxia with treatment planning and prognosis. In general, the accumulation of a hypoxic sensitive probe, such as [^18^F]F-FMISO, in a tumor has been related to poor prognosis and response to treatment (Horsman et al., 2012). The available clinical data show that the measurement and imaging of hypoxic regions could be a determinant factor in the identification of poor prognosis and benefit associated from hypoxia-targeted treatment (Table 1).

**Table 1:** Summary of clinical imaging findings and recommendations for the use of most common hypoxia tracers.

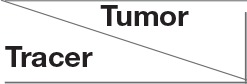	**[^18^F]FMISO**	**[^18^F]F-HX4**	**[^18^F]FAZA**	**[^18^F]FETNIM**	**[^18^F]F-EF5**	**[^18^F]F-RP170**	**^64^CuCu(ATSM)**
Brain							
Head and neck							
Breast							
Sarcoma							
Lung							
Lymphoma							
Renal							
Liver							
Colorectal							
Bladder							
Cervical							
Prostate							

*Adapted from Fleming et al. (2014)*.

*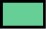 Yes, good clinical data obtained*.

*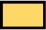 Recommended favorable preclinical/metabolic data*.

*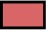 Not recommended, unfavorable preclinical/metabolic data*.

*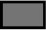 No, poor clinical data*.

The information obtained during hypoxia imaging can be translated to treatment following two main strategies: tailoring the radiotherapy and planning by hypoxia therapeutics. The benefit of the use of these techniques has been clinically demonstrated in a large sample of patients with head and neck cancers (Overgaard, 2011). A tailored radiotherapy planning aids in designing a patient-specific plan depending on the tumor characteristics and the presence of reduced pO_2_ areas. It has long been known that hypoxic tumors need at least 2–3 times the amount of radiation to be effective when compared to normoxic tissue (Gray et al., 1953). As a consequence, the radiotherapy plan can be modified through dose escalation to the gross tumor volume or dose painting (only in the volumes identified as hypoxic) (Horsman et al., 2012). The concept of dose painting is promising and was made possible thanks to advances in dose distribution applications by intensity-modulated radiation therapy (IMRT) (Galvin and De Neve, 2007) which allows for the delivery of a higher dose to sub-volumes previously identified as hypoxic. However, this technique has not demonstrated its clinical applicability to date and it has a number of disadvantages such as the possible change in hypoxic regions due to dynamic metabolic processes, in addition to the insufficient spatial resolution of the imaging methods used, that can result in missed areas (e.g., the PET imaging resolution is in the 1–2 mm scale while a hypoxic region can be as small as on a μm scale: see Figure 2B for characteristics on different imaging modalities; Geets et al., 2013; Long and Wong, 2014).

The other basic strategy is using hypoxia as a therapeutic target, mainly by the use of radiosensitizers (nimorazole, misonidazole, metronidazole) or cytotoxins (tirapazamine, evofosfamide). Radiosensitizers are hypoxia-activated prodrugs (HAP) which are inhibited/deactivated under the presence of oxygen and active under hypoxia by reduction, commonly by oxidoreductase enzymes, generating radical species that are accumulated in the cell causing toxicity (Wang et al., 2012). Cytotoxins are used as neoadjuvant and have demonstrated to be able to control metastasis and to provide cooperative therapy when used alongside radiotherapy (Hong et al., 2016).

## Author contributions

All authors listed have made a substantial, direct and intellectual contribution to the work, and approved it for publication.

### Conflict of interest statement

The authors declare that the research was conducted in the absence of any commercial or financial relationships that could be construed as a potential conflict of interest.

## Footnotes

^1^http://www.who.int/mediacentre/factsheets/fs355/en/.
