# Corrigendum: Oxygen Sensing, Hypoxia Tracing and *in Vivo* Imaging with Functional Metalloprobes for the Early Detection of Non-communicable Diseases

**DOI:** 10.3389/fchem.2018.00512

**Published:** 2018-10-23

**Authors:** Vincenzo Mirabello, Fernando Cortezon-Tamarit, Sofia I. Pascu

**Affiliations:** Department of Chemistry, University of Bath, Bath, United Kingdom

**Keywords:** oxygen sensing, molecular imaging, hypoxia, metals in medicine, FRET

In the original article, there was a mistake in the legend for Figure 1 as published. The figure was not reprinted from (Hauge et al., 2017) but from (Horsman et al., 2012). The corrected legend appears below.

Figure 1. Schematic representation of tumor cells distribution under chronic **(A)** and acute **(B)** hypoxia. In a diffusion-limited chronic hypoxia condition **(A)** the oxygen molecules diffusing from the vessels are used by normoxic cells which result in a decrease of pO_2_ **(C)**. The reduced level of molecular oxygen triggers the proliferation of hypoxic cells at the periphery of the blood vessels. When the vessels are functionally compromised, all the cells around the capillary become acutely hypoxic. Representative flows of pO_2_ over time in the occluded region are shown for comparison **(D)**. Adapted with permission from Horsman et al. (2012).

Additionally, there was a small mistake in Figure 6 as there is a missing a methylene group. The corrected Figure 6 appears below.

**Figure 6:**
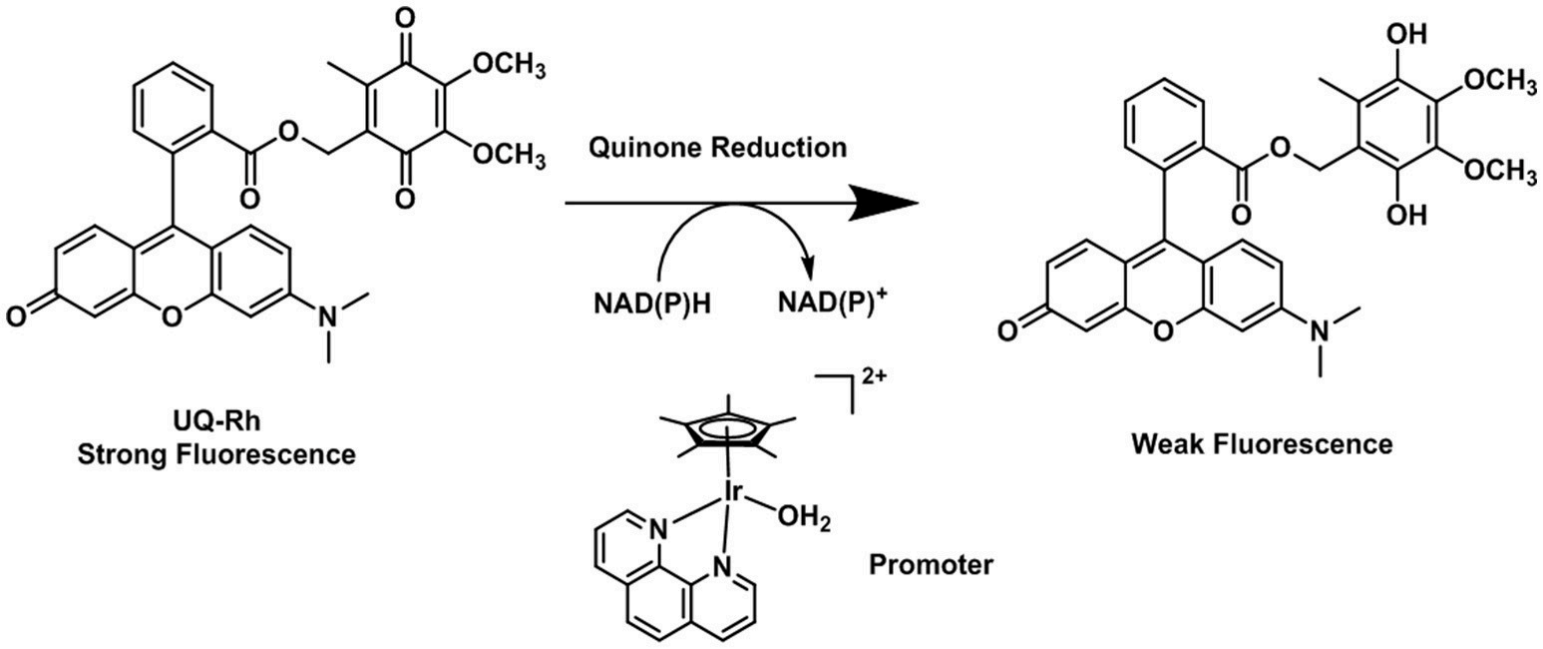
Reduction process of the fluorescent quinone derivative UQ-Rh by NAD(P)H (Komatsu et al., 2014).

In the original manuscript there was one error in Horsman et al. (2012) reference. The title read “Imaging hypoxia to improve radiotherapy outcome” and has now been corrected. Also page number range should be 674–687. The corrected wording for these references appear in the Reference List.

The authors apologize for these errors and state that this does not change the scientific conclusions of the article in any way.

The original article has been updated.

## Conflict of interest statement

The authors declare that the research was conducted in the absence of any commercial or financial relationships that could be construed as a potential conflict of interest.
